# Impact of Ambient Air Pollution on Cardiovascular Diseases in Low- and Lower-Middle-Income Countries: A Systematic Review and Meta-Analysis

**DOI:** 10.5334/gh.1545

**Published:** 2026-03-30

**Authors:** Marvellous Adeoye, Natalie Evans, Shadi Rahimzadeh, Shreya Shrikhande, Sean Taylor, Pablo Perel, Anoop Shah, Mariachiara Di Cesare, Mark R. Miller

**Affiliations:** 1 Institute of Public Health and Wellbeing, University of Essex, Colchester, United Kingdom; 2 Department Of Science and Public Health, World Heart Federation, Geneva, Switzerland; 3 Department of Non-Communicable Disease Epidemiology, London School of Hygiene & Tropical Medicine, London, United Kingdom; 4 Department of Cardiology, Imperial College NHS Trust, London, United Kingdom; 5 Institute for Neuroscience and Cardiovascular Research, University of Edinburgh, Edinburgh, United Kingdom

**Keywords:** particulate matter, nitrogen dioxide, hospital admissions, mortality, developing countries, LMICs

## Abstract

Air pollution contributes to over 8.1 million deaths annually, predominantly from cardiovascular causes. The burden of air pollution is significantly higher in low-income countries (LICs) and lower middle-income countries (LMICs), yet most air pollution research is performed in higher-income countries. Our objective was to systematically review the association between exposure to ambient air pollution and cardiovascular disease (CVD) in LICs and LMICs. PubMed and Global Health databases were systematically searched for studies that explore associations between daily increases in gaseous (SO_2_, NO_2_, CO, O_3_) and particulate matter (PM) air pollutants with CVD mortality and hospital admission in adults. Studies were assessed for risk of bias based on outcome validity, exposure measurement quality, and confounder adjustment. A random-effects model was used to estimate overall and per-pollutant risks from short-term exposure studies, standardised to 10 μg/m^3^ increments. Of 1329 articles screened, 48 met the inclusion criteria, of which 22 included a measure of relative risk suitable for meta-analysis. Short-term exposure to PM_2.5_ and PM_10_ were associated with increased combined mortality and hospital admission with 0.53% (95% CI: 0.31%–0.75%) and 1.68% (95% CI: 0.17%–3.21%) increase per 10 μg/m^3^, respectively. NO_2_ showed a 0.66% increase (95% CI: 0.36%–0.97%) per 10 μg/m^3^. This systematic review highlights the limited evidence on air pollution and CVD in LICs and LMICs. Nonetheless, this meta-analysis found positive associations between several ambient air pollutants and cardiovascular hospital admissions and mortality. There is the vital need for further research in underrepresented regions, particularly on the effects of long-term exposure, in order to establish the true burden of air pollution on cardiovascular health in regions where air pollution is frequently high, or access to healthcare is limited.

## 1. Introduction

Air pollution (both indoor and outdoor), which contributed to over eight million deaths globally in 2021, is considered one of the most worrying global health emergencies (1,2). It is both the second largest modifiable risk factor for mortality and the greatest risk factor for disability-adjusted life years (DALYs) (3). Almost 90% of global ambient air pollution related deaths occur in low- and lower-middle-income countries (LICs and LMICs) (4). The disproportionate burden of air pollution-related cardiovascular disease in LICs and LMICs stems from multiple factors. While higher ambient pollution concentrations play a significant role, there are also important considerations regarding potential differences in air pollution composition and population demographics that may not be fully captured in current global models. Understanding whether the same exposure produces similar or different cardiovascular effects across diverse settings is critical for accurate burden assessment.

Ambient (outdoor) air pollution can arise from many sources and consequently contains a large mixture of thousands of chemicals, which vary spatially and temporally depending on source, dispersion, atmospheric reactions and meteorological conditions (5). Criteria air pollutants that affect health include particulate matter (PM) of varying size, nitrogen dioxide (NO_2_), ozone (O_3_), sulfur dioxide (SO_2_) and carbon monoxide (CO). Fine particulate matter (PM with aerodynamic diameter ≤2.5 μm; ‘PM_2.5_’) is the air pollutant that is most consistently associated with adverse health effects especially in organ systems beyond the lung.

In 2021, cardiovascular diseases (CVDs) were responsible for 52% of the total DALYs attributable to ambient PM_2.5_ exposure (3). Both short- and long-term exposure to air pollution is associated with increased risk of CVD (6,7), including cardiovascular outcomes such as myocardial infarction (MI) (8,9,10), stroke (11,12), and heart failure (HF) (13,14). The causality of these observations are supported by a substantial body of mechanistic research that show that inhaled air pollutants, especially PM, induce cardiovascular dysfunction through multiple mechanisms, such as oxidative stress, inflammation, endothelial dysfunction, thrombosis, autonomic imbalance, and acceleration of the growth and instability of atherosclerosis plaques (15,16).

The majority of epidemiological studies on air pollution have been conducted in high-income countries (HICs) (17,18), and may not be generalisable to LICs and LMICs where sources of air pollution and population demographics and diseases differ. For example, current global estimates of the health impact of air pollution in LICs and LMICs, including those from the Global Burden of Disease (GBD) study, rely heavily on exposure-response functions derived predominantly from HICs. These estimates often apply concentration-response relationships from HICs to pollution levels and demographic profiles in LMICs, with limited validation from local studies. This extrapolation approach, while practical given data limitations, introduces uncertainty in burden estimates. Removing or lowering risk factors is key to the prevention of CVD (19,20) and is especially important in LMIC settings given that the availability of treatment may be limited.

Despite recognition of the disproportionate burden of both air pollution exposure and CVD suffered by LICs and LMICs, the evidence base remains relatively limited (21,22). Existing reviews group LMICs with upper-middle-income countries (7,18) or focus only on particulate matter (23). Another notable gap is the absence of pooled estimates of exposure-response relationships, which hinders an adequate burden assessment and means to direct public health policy. Here, we aimed to systematically review and synthesise the evidence on associations between ambient air pollution exposure and cardiovascular outcomes in, specifically, LICs and LMICs. Our review has been designed to include all criteria air pollutants (PM_2.5_, PM_10_, SO_2_, NO_2_, CO, and O_3_) and both cardiovascular mortality and hospitalisation. The review aims not only to establish and quantify these effects of air pollution in these regions, but also to identify research gaps that are needed to ascertain full burden on health and help guide air quality interventions that could improve cardiovascular health.

## 2. Methods

### 2.1 Search strategy and selection criteria

The systematic review and meta-analysis protocol was registered with PROSPERO (registration CRD42023484929) and adhered to the Preferred Reporting Items of Systematic Reviews and Meta-Analysis (PRISMA) guidelines (24).

PubMed and Global Health databases were systematically searched for relevant resources. The search terms were a pairwise combination of ‘air pollution’ (including particulate matter (PM_2.5_ and PM_10_ (particles with aerodynamic diameter ≤10 μm)), SO_2_, NO_2_, CO, O_3_) and ‘cardiovascular disease’, ‘myocardial infarction’, ‘stroke’, and ‘heart failure’ (HF), selected to capture both overall CVD outcomes and specific CVDs with well-established links to ambient air pollution exposure. These terms were combined using AND, and OR, and some were expanded, especially those with multiple synonyms, to include all pertinent articles. Adjustments to the search were made as necessary, by the specific requirements of the database used. The search spanned the inception of the database to June 2024 (for full search strategy, see **Supplementary Material**).

Studies were included if published in English, conducted in LICs or LMICs as from the 2024 World Bank definition (**Figure S1**), and reported associations between ambient air pollution and the aforementioned CVD endpoints. We included studies of all age groups with no restrictions on population demographics. This review focused on ambient air pollution; studies examining indoor air pollution or non-ambient occupational air pollution exposure were excluded. We included both short-term exposure studies (examining acute exposure over days to weeks) and long-term exposure studies (examining chronic exposure over months to years). Various methodological approaches were eligible—including time-series, case-crossover, cohort studies, cross-sectional, ecological, cost analysis, comparative risk assessment designs, and studies using the World Health Organization (WHO) Health Impact Assessment model (e.g., AirQ+), which estimates health impacts of air pollution using standardised assumptions and software tools—as long as they reported quantitative associations between ambient air pollution and cardiovascular outcomes. Summaries, editorials, short commentaries, conference proceedings, non-peer-reviewed studies, and full texts that were not available online were all excluded.

### 2.2 Screening and data extraction

Results from the search were entered into Endnote, and duplicates removed. Rayyan was used to screen the papers. Titles/abstracts and full text-screening, and data extraction process were conducted independently by M.A. and S.R. For each article, meta-data information was extracted via a predefined data-extraction tool previously used (22). In summary, the following were extracted: (1) study characteristics (first author, publication year, study design, country, and data source); (2) ambient air pollution measurement and measurement tools (e.g., fixed monitoring stations, satellite-derived estimates, or personal monitors) and period of exposure (short-term or long-term); and (3) CVD admission and mortality (22).

Any disparities between the reviewers were deliberated, and if necessary, resolved by a third reviewer (S.S.).

### 2.3 Risk of bias assessment

The risk of bias assessment tool adapted for this study has been previously reported (9,22,25). This evaluation covers three components: validity of the occurrence of CVD (rated 0 or 1), quality of air pollutant measurements (rated 0 or 1), and the degree of confounder adjustment (rated 0, 1, 2, or 3). Studies were rated as good quality (best scores in all domains), average (mixed scores), or low quality (any domain scored zero). (For full description, see **Supplementary Material**.)

### 2.4 Data analysis

For the meta-analysis, we included only studies reporting relative risk (RR), hazard ratio (HR), or odds ratio (OR) for lag zero or lag one day. Only one study provided cumulative lags (0–1) which was used as the shortest lag. RR were pooled for a standardised increment in pollutant concentration, with all concentrations standardised to a 10 μg/m^3^ increment. For this standardisation, we used the following conversions specific for each pollutant: one part per billion (ppb) was converted to 1.88 μg/m^3^ for NO_2_, 2.62 μg/m^3^ for SO_2_, and 1.96 μg/m^3^ for O_3_. One part per million (ppm) was converted to 1145 μg/m^3^ for CO (under the assumption of standard temperature and pressure of 25 °C and 1 atm) (26).

In line with established methodological practices in air pollution meta-analyses (14), we assumed a linear relationship between exposure and outcome for standardisation purposes. Standardised risk estimates were calculated for each study using the following equation (14):


1 \[RR{\,_{\left({{\mathrm{standardised}}} \right)}} = \,\,RR{\,^{{\mathrm{Increment }}\left({{\mathrm{1}}0} \right)/{\mathrm{Increment}}\left({{\mathrm{original}}} \right)}}\]

To ensure consistency across studies and reduce heterogeneity from differing lag structures, we extracted effect estimates for the shortest lag available (typically lag zero or lag 1) to assess overall risk estimates.

In studies reporting multiple health CVD outcomes (e.g., stroke, MI, HF) under exposure to the same pollutant and categorised by the same outcome type (admission, mortality), we conducted an internal meta-analysis to derive a single pooled effect. This pooled estimate was then used as a representative measure for that study in the overall meta-analysis of CVD admissions and mortality. For studies reporting both specific CVD outcomes and a broader overall CVD outcome for the same pollutant and endpoint, we exclusively used the overall CVD admission or mortality outcome to avoid double-counting and ensure consistency in the pooled analysis.

Funnel plots were used to examine publication bias and assessed for asymmetry using Egger’s regression test; correction for asymmetry was performed using the trim and fill method (27).

A sensitivity analysis was conducted excluding 1) ORs; 2) studies from Iran (which represented the majority of included studies).

Because of the diversity in study designs, methods, lag exposures, and geographical and population differences, we used a random-effects model to account for within and between study heterogeneity. Heterogeneity was examined using the standard *I*^2^ test. Statistical significance was taken as two-sided p < 0.05. Analysis was performed using R Software (version 4.4.2).

## 3. Results

### 3.1 Study overview

**Figure 1:**
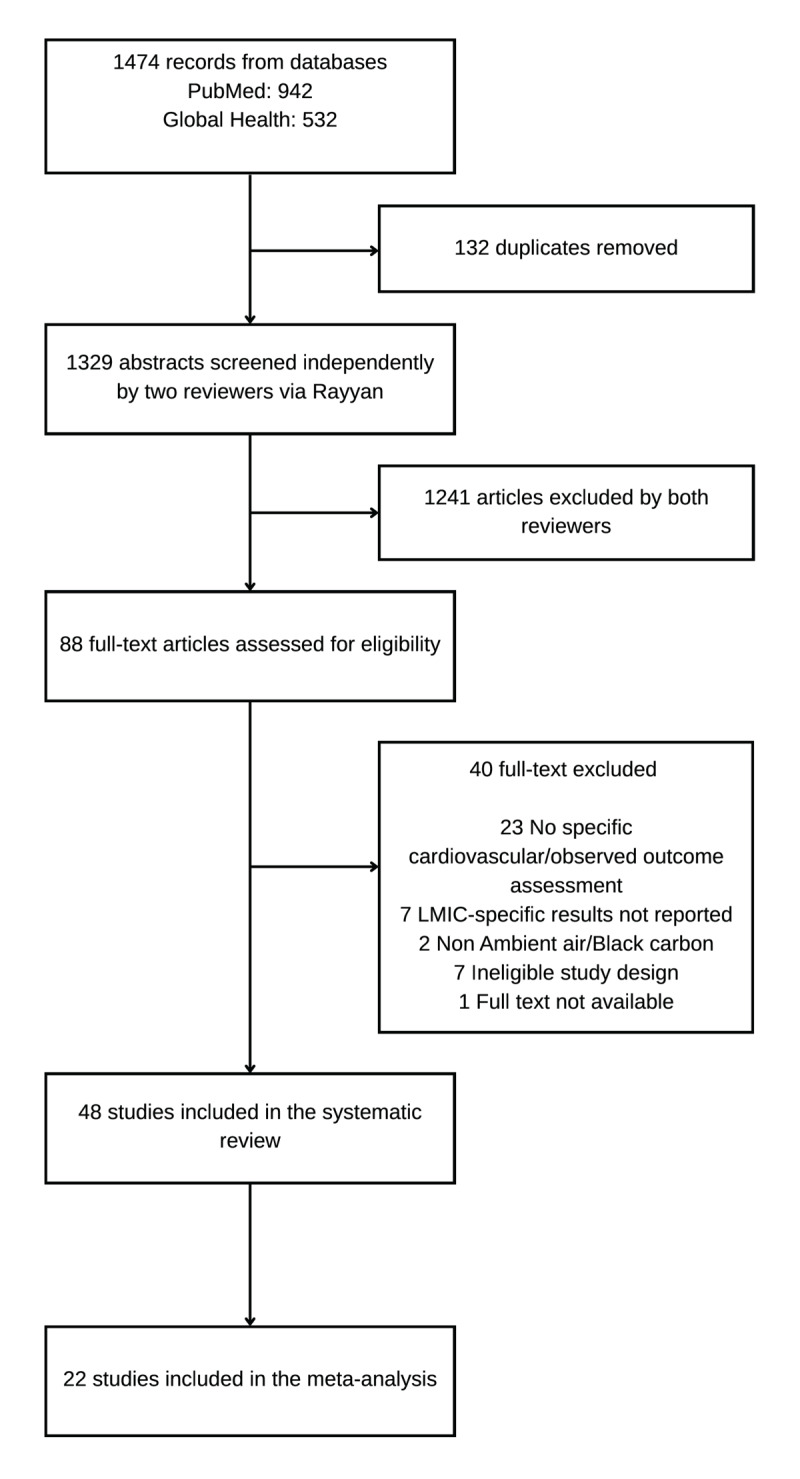
PRISMA flow chart.

The initial literature search identified 1474 research articles. After removing duplicates, the articles were reduced to 1329. Following titles/abstracts and full-text review, 48 studies met the criteria for inclusion (Figure 1 and Table 1). The majority of studies were conducted in Iran (n = 38), followed by Vietnam (n = 3) and Bangladesh (n = 3). Indonesia, India, Nepal, and Lebanon had one study each (**Figure S2**). This includes one multi-country study (28) covering France, Iran, and Italy, from which only the Iran data were extracted. There were more short-term studies (n = 39) than long-term studies (n = 9). Time series and case-crossover studies were the most frequently used study designs (n = 23), followed by studies using the WHO Health Impact Assessment model (n = 14), cross-sectional studies (n = 5) and cohort studies (n = 3). Furthermore, three studies used cost analysis (n = 1), ecological (n = 1), and comparative risk assessment (n = 1) designs. Of the 48 studies included in the review, the majority (31; 64.6%) were of good or average methodological quality, with the most common limitations being inadequate confounder adjustment and insufficient air pollution measurement quality.

**Table 1:** Baseline characteristics of included studies.


AUTHOR	YEAR	COUNTRY	DATA SOURCE	DURATION	DESIGN	OUTCOME	POLLUTANTS	EXPOSURE ASSESSMENT	ROB ASSESSMENT

(63) (*)	2017	Iran	Iran statistical centre	Short term	Cross-sectional	admission	SO_2_	Ground monitoring stations	Low

(64) (*)	2017	Iran	Hospital database	Short term	Cross-sectional	admission	SO_2_, NO_2_, NO, NO_x_, CO, O_3_, PM_10_	Ground monitoring stations	Average

(33) (*)	2019	Iran	Hospital database	Short term	Time series	admission	NO_2_, NO, O_3_	Ground monitoring stations	Good

(65) (*)	2005	Iran	Hospital database	Long term	Time series	admission	SO_2_, NO_2_, CO, O_3_, PM_10_	Ground monitoring stations	Good

(66)	2022	Vietnam	Hospital database	Short term	Time series	admission	PM_10_, NO_2_	Ground monitoring stations	Average

(37) (*)	2016	Vietnam	Hospital database	Short term	Time series	admission	NO_2_, PM_10_, SO_2_	Ground monitoring stations	Average

(67) (*)	2017	Iran	Hospital database	Short term	Time series	admission	SO_2_, CO_2_, NO_2_, O_3_, PM_2.5_, PM_10_	Ground monitoring stations	Good

(32) (*)	2023	Iran	Clinical registry	Short term	Time series	admission	CO	Ground monitoring stations	Good

(34) (*)	2019	Iran	Hospital database	Short term	Case crossover	admission	O_3_, SO_2_, NO_2_, CO, PM_10_, PM_2.5_	Ground monitoring stations	Average

(68)	2022	Indonesia	Survey data	Long term	Cross-sectional	admission	PM_2.5_	Satellite monitoring	Low

(38) (*)	2019	Bangladesh	Hospital database	Short term	Case crossover	admission	PM_2.5_	Ground monitoring stations	Average

(35) (*)	2023	Iran	Hospital database	Short term	Case crossover	admission	PM_2.5_, PM_10_	Ground monitoring stations	Average

(69)	2017	Iran	Hospital database	Short term	HIA, WHO AirQ	mortality	O_3_	Ground monitoring stations	Low

(70)	2015	Iran	WHO AirQ	Short term	HIA, WHO AirQ	mortality	O_3_	Ground monitoring stations	Low

(39) (*)	2021	Bangladesh	Hospital database	Short term	Time series	mortality, admission	PM_2.5_	Ground monitoring stations	Average

(71)	2017	Iran	WHO AirQ	Short term	HIA, WHO AirQ	mortality	PM_2.5_, PM_10_	Ground monitoring stations	Low

(72)	2016	Iran	WHO AirQ	Short term	HIA, WHO AirQ	mortality	PM_2.5_	Ground monitoring stations	Low

(73)	2020	Iran	WHO AirQ	Long term	HIA, WHO AirQ	mortality	PM_2.5_	Ground monitoring stations	Low

(36) (*)	2019	Iran	Hospital database	Short term	Case crossover	admission	PM_2.5_, PM_10_, SO_2_, O_3_ CO, NO_2_	Ground monitoring stations	Average

(28) (*)	2019	France, Iran, Italy	WHO AirQ	Short term	HIA, WHO AirQ	mortality, admission	PM_10_, PM_2.5_, O_3_	Ground monitoring stations	Low

(74) (*)	2023	Iran	Hospital database	Short term	Time series	admission	O_3_, CO, NO_2_, SO_2_, PM_10_, PM_2.5_	Ground monitoring stations	Average

(75) (*)	2022	Iran	Hospital database	Short term	Time series	admission, mortality	SO_2_, NO_2_, CO, O_3_	Ground monitoring stations	Good

(76)	2021	Iran	Hospital database	Short term	Time series	admission	CO, O_3_, PM_2.5_, NO_2_, SO_2_	Ground monitoring stations	Good

(77) (*)	2012	Iran	Hospital database	Short term	Cross-sectional	admission	SO_2_, CO, NO	Ground monitoring stations	Average

(30) (*)	2021	Iran	WHO AirQ	Long term	Time series	mortality	PM_2.5_	Ground monitoring stations	Low

(78)	2018	Iran	WHO AirQ	Short term	HIA, WHO AirQ	mortality	PM_10_, PM_2.5_	Ground monitoring stations	Low

(79)	2020	Vietnam	Hospital database	Short term	Case crossover	admission	PM_10_, PM_2.5_, SO_2_, CO, NO	Ground monitoring stations	Good

(80)	2016	Iran	WHO AirQ	Short term	HIA, WHO AirQ	mortality	PM_10_, NO_2_, O_3_	Ground monitoring stations	Low

(81)	2019	Iran	Municipal records	Long term	Cost analysis	mortality	PM_2.5_	Ground monitoring stations	Low

(82)	2014	Iran	WHO AirQ	Short term	HIA, WHO AirQ	admission	PM_10_	Ground monitoring stations	Average

(83)	2022	Iran	WHO AirQ	Short term	HIA, WHO AirQ	Mortality, admission	PM_2.5_, PM_10_	Ground monitoring stations	Low

(84)	2017	Iran	WHO AirQ	Short term	HIA, WHO AirQ	mortality	PM_10_	Ground monitoring stations	Low

(85) (*)	2018	Iran	Hospital database	Short term	Ecological	mortality	PM_10_, O_3_, NO_2_, SO_2_, CO	Ground monitoring stations	Good

(86)	2024	Bangladesh	Registry data	Long term	Cohort	mortality	PM_2.5_	Satellite monitoring	Average

(87)	2021	Iran	Cohort data	Long term	Cohort	mortality	PM_2.5_	Satellite monitoring	Average

(88)	2015	Iran	National registry	Short term	Cross-sectional	mortality	O_3_, CO, NO_2_, SO_2_, PM_10_	Ground monitoring stations	Average

(89)	2018	Iran	WHO AirQ	Short term	HIA, WHO AirQ	mortality, admission	O_3_	Ground monitoring stations	Low

(90)	2016	Iran	WHO AirQ	Short term	HIA, WHO AirQ	mortality	SO_2_, NO_2_	Ground monitoring stations	Low

(91)	2022	India	Survey/registry data	Long term	Cohort	mortality	PM_2.5_	Ground monitoring stations	Good

(92)	2017	Iran	Registry data	Long term	Comparative risk assessment	mortality	PM_2.5_	Ground monitoring stations	Average

(93)	2017	Nepal	Hospital database	Short term	Case crossover	admission	PM_10_	Ground monitoring stations	Good

(94)	2020	Iran	Hospital database	Short term	Time series	admission	CO, O_3_, SO_2_, NO_2_, PM_10_	Ground monitoring stations	Good

(29) (*)	2021	Iran	Hospital database	Short term	Time series	admission	PM_2.5_	Ground monitoring stations	Average

(31) (*)	2019	Iran	Hospital database	Short term	Time series	admission	PM_10_, O_3_, NO_2_	Ground monitoring stations	Average

(40) (*)	2015	Lebanon	Hospital database	Short term	Time series	admission	PM_2.5_, PM_10_	Ground monitoring stations	Good

(95)	2024	Iran	WHO AirQ	Short term	HIA, WHO AirQ	mortality	NO_2_, O_3_, SO_2_	Ground monitoring stations	Low

(96) (*)	2018	Iran	Hospital database	Short term	Case crossover	admission	CO, O_3_, NO_2_, PM_10_, SO_2_	Ground monitoring stations	Good

(97)	2020	Iran	Municipal records	Short term	Time series	mortality	PM_2.5_	Ground monitoring stations	Good


Note: (*) studies included in the meta-analysis, Air quality modelling software (WHO AirQ). WHO, World Health Organization; HIA, Health Impact Assessment; AirQ, Air Quality Health Impact Assessment tool.

### 3.2 Meta-analysis

Overall CVD effects were obtained for four studies (**Figure S3**) through internal meta-analyses combining HF admission and MI admission for PM_2.5_ (29); ischemic heart disease (IHD) mortality and stroke mortality for PM_2.5_ (30); IHD admission and MI admission for CO, NO_2_, O_3_, and SO_2_ separately (31); HF admission, IHD admission and stroke admission for CO (32). Overall CVD admission was provided as an outcome and used instead of other CVD specific outcomes for two studies (31,33). **Figure S3** presents these combined within-study effect estimates that were subsequently used in our main meta-analysis.

The detailed pooled effect size RRs of pollutants for a 10 μg/m^3^ increase along with p-values and heterogeneity are given in Table 2. The meta-analysis included a total of 22 studies and 101 data points investigating air pollutant-outcome pairs. Most of the studies (21; 88 estimates) examined the association between air pollutants and hospital admission for CVD, while only five studies (13 estimates) investigated associations with mortality.

**Table 2:** Meta-Analysis results.


CATEGORY	RR	95% CI LOWER	95% CI UPPER	NUMBER OF STUDIES	NUMBER OF DATA POINTS	I^2^

**CO**						

mortality + admission	1.0001	1.0001	1.0001	12	14	84.8%

admission	1.0001	1.0001	1.0001	11	13	86.0%

mortality	–	–	–	–	–	–

**NO_2_**						

mortality + admission	1.0066	1.0036	1.0097	12	15	56.1%

admission	1.0062	1.0026	1.0099	11	13	51.0%

mortality	1.0108	1.0008	1.021	2	2	85.0%

**NO**						

mortality + admission						

admission	1.002	0.9987	1.0053	3	3	28.5%

mortality	–	–	–	–	–	–

**O_3_**						

mortality + admission	0.9995	0.9988	1.0002	12	15	84.3%

admission	0.999	0.9985	0.9995	10	12	83.5%

mortality	1.0038	1.0007	1.0069	3	3	47.7%

**PM_10_**						

mortality + admission	1.0168	1.0017	1.0321	13	19	88.1%

admission	1.0191	1.0017	1.0368	12	17	87.2%

mortality	1.0032	0.9984	1.008	2	2	95.1%

**PM_2.5_**						

mortality + admission	1.0053	1.0031	1.0075	12	18	74.3%

admission	1.0046	1.0025	1.0067	11	15	72.1%

mortality	1.0384	0.9813	1.0988	3	3	86.8%

**SO_2_**						

mortality + admission	1.0015	0.9986	1.0044	13	16	34.4%

admission	1.0022	0.9991	1.0053	12	14	34.3%

mortality	0.9976	0.9909	1.0044	2	2	25.6%


**Figure 2:**
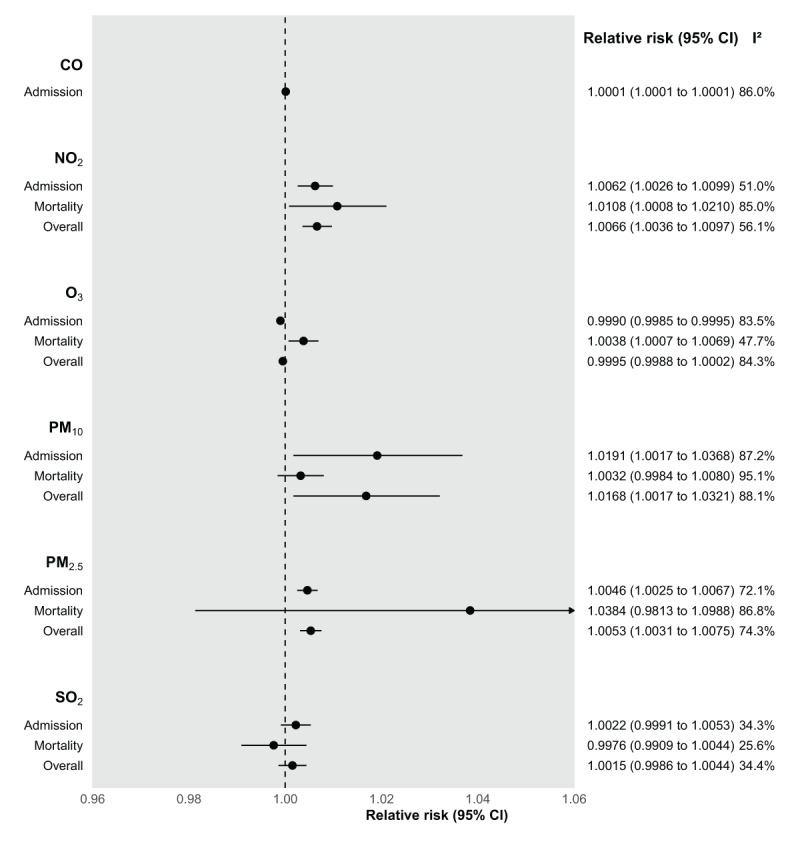
Forest plot of pooled relative risks for cardiovascular outcomes (per 10 µg/m^3^ increment).

Daily exposure to PM_2.5_ and PM_10_ were both associated with increased combined hospital admission and CVD mortality with an RR of 1.0053 (95% Confidence Interval (CI): 1.0031–1.0075; *I*^2^ = 74.3%) and 1.0168 (95% CI: 1.0017–1.0321; *I*^2^ = 88.1%) per 10 μg/m^3^ increase in daily concentrations respectively (Figure 2). When stratified by outcome (admission or mortality), there was strong evidence of association between exposure to 10 μg/m^3^ of PM_2.5_ and hospital admissions for CVD with an RR of 1.0046 (95% CI: 1.0025–1.0067; *I*^2^ = 72.1%), while the same level of exposure to PM_10_ showed strong evidence of association with an RR of 1.019 (95% CI: 1.0017–1.0368; *I*^2^ = 87.2%) for hospital admission. The RR for mortality was larger for PM_2.5_ (RR = 1.0384; 95% CI: 0.9813–1.0988; *I*^2^ = 86.8%) compared to PM_10_ (RR = 1.0032; 95% CI: 0.9984–1.008; *I*^2^ = 95.1%), although neither were statistically significant due to the high variability of risk estimates, especially for PM_2.5_ mortality.

Exposure to gaseous pollutants was reported in approximately half of the studies included in the analysis. NO_2_ generally had the strongest positive associations with an overall RR of cardiovascular outcomes of 1.0066 (95% CI: 1.0036–1.0097; *I*^2^ = 56.1%), 1.0062 (95% CI: 1.0026–1.0099; *I*^2^ = 51.0%) for admission alone and 1.0108 (95% CI: 1.0008–1.0210; *I*^2^ = 85.0%) for mortality alone, per 10 μg/m^3^ increase in NO_2_. O_3_ was associated with an increased RR of mortality of 1.0038 (95% CI: 1.0007–1.0069; *I*^2^ = 47.7%) per 10 μg/m^3^, however there was no association with CVD admission or the overall CVD outcome. Increases in levels of CO were associated with an RR of 1.0001 (95% CI: 1.0001–1.0001; *I*^2^: 86%) in hospital admissions per 10 μg/m^3^. No significant associations were detected for SO_2_ or NO with either CVD mortality or admission.

Publication bias (Egger’s test for asymmetry p < 0.05) was observed only for NO_2_ (p = 0.0083) and CO (p < 0.001) (**Table S1**). Adjustment for asymmetry with the trim and fill method did not alter the effect direction or effect size.

### 3.3 Sensitivity analysis

A sensitivity analysis was conducted excluding three studies (34,35,36) which reported OR for hospital admission rather than RR, to account for any bias introduced with the assumption OR approximation to RR. While the pattern of effects remained the same, the effect size for daily exposure to PM_10_ and PM_2.5_ were both associated with reductions in the RR of combined CVD mortality and hospital admissions in the sensitivity analysis, with an RR of 1.0065 (95% CI: 0.9941–1.0189; *I*^2^ = 87.1%) and 1.0056 (95% CI: 1.0026–1.0087; *I*^2^ = 48.8%) per 10 μg/m^3^ increase in daily concentrations, respectively (**Figure S4**). These changes were driven by reductions in the effect size for hospital admissions, bringing those associations closer to the null. For the gaseous pollutants, the only effect sizes impacted by this sensitivity analysis were related to NO_2_, where the RR for combined mortality and hospital admissions reduced slightly to 1.0065 (95% CI: 1.0037–1.0094; *I*^2^ = 58.1%). No other effect sizes were impacted by this sensitivity analysis.

An additional analysis was performed to assess whether the predominance of Iranian studies (38 out of 48 studies) might have biassed our overall findings, and to determine if the observed associations were consistent across different geographical settings. This meta-analysis was conducted on five studies (28, 37–40) (17 data points) after excluding all data from Iran (**Figure S5**). PM_10_ showed a smaller but less heterogeneous effect in the non-Iranian studies (RR = 1.0062; 95% CI: 1.0042–1.0081; *I*^2^ = 0%) versus Iranian studies (RR = 1.0221; 95% CI: 1.0022–1.0424; *I*^2^ = 87.5%). For PM_2.5_, the non-Iranian studies had a lower but still significant effect estimate (RR = 1.0041; 95% CI: 1.0020–1.0061; *I*^2^ = 0%) compared to Iranian studies (RR = 1.0091; 95% CI: 1.0041–1.0142; *I*^2^ = 81.5%). Notably, the non-Iranian studies demonstrated markedly lower heterogeneity (*I*^2^ = 0–6.4%) compared to the Iranian studies (*I*^2^ = 79.5–87.5%). Due to the limited number of non-Iranian studies, separate analyses by outcomes were not feasible. In many cases, only one or two studies contributed data, although most of these individual study findings were consistent with the overall associations observed in the pooled analyses.

## 4. Discussion

This systematic review and meta-analysis find that short-term exposure to ambient air pollution, particularly for PM_2.5_, PM_10_, and NO_2_, is associated with CVD outcomes in LICs and LMICs. These results complement global findings that air pollution is linked to cardiovascular morbidity and mortality, extending these findings beyond these data, which were predominantly from middle- to high-income countries, to lower income nations. While statistically significant associations were found, this review highlights the paucity of data from different countries that is needed to ascertain the true burden of air pollution in low-income nations.

Our findings build on the previous global meta-analysis by Shah et al. in 2013 and 2015 (12,14), which linked air pollution to HF and stroke, by focusing specifically on LICs and LMICs. For CVD hospital admissions, the pooled PM_2.5_ effect observed here (0.46% increase per 10 μg/m^3^) is lower than the 1.01% increase reported by Shah et al., but slightly higher than 0.26–0.29% reported from studies using data from more than 180 cities in China, an upper-middle income country (41,42). By comparison, data from the USA, a high income country, found that a 10 μg/m^3^ increase in PM_10–2.5_ was associated with a 0.69% increase in same-day cardiovascular hospital admissions (43). When examining mortality outcomes, our estimates (3.84% increase per 10 μg/m^3^) exceed those reported in previous short-term exposure systematic reviews from global (0.84%) and China-specific (0.63%) settings (44,45).

Larger effects are typically observed for long-term cohort studies in HICs. For instance, in a US cohort study (7.5 million person-years) there was a considerably higher increase (14%, 95% CI: 2–27%) in stroke mortality and in IHD mortality (16%, 95% CI: 9–22%) per 10 μg/m^3^ PM_2.5_ (46). This greater risk ratio in long-term studies reflects that admissions and mortality outcomes will likely capture any long-term effects of air pollution on disease progression as well as the acute effects of air pollution on exacerbation of CVD that leads to a cardiovascular event. Longitudinal cohort studies are more common in HICs due to factors that include the availability of long-term air pollution monitoring data from stationary monitoring networks, comprehensive medical records data systems, and the greater availability of resources required for prospective participant follow-up over many years. The limited number of long-term exposure studies in LICs/LMICs represents a critical research gap that needs to be addressed to establish the full burden of chronic air pollution exposure in these settings.

We observed that NO_2_ generally had the strongest positive associations among gaseous pollutants. NO_2_ is a gas with oxidative properties that induces pulmonary inflammation and oxidative stress; effects that can be transmitted to the circulation to impair cardiovascular function through multiple mechanisms, especially with long-term exposure (47). NO_2_ is also a marker of traffic-related air pollution, emissions of which also include semi-volatile species and particulate matter, especially ultrafine particles, which are infrequently assessed in epidemiological studies; there may therefore be a degree of ‘effect transfer’ between closely correlated pollutants (48). Nonetheless, rapidly urbanising LMIC settings will see increasing vehicle numbers and, frequently, an older vehicle fleet that generates significant emissions with less stringent emission controls. A greater commitment towards targeted interventions to reduce traffic emissions in LICs/LMICs could therefore lead to significant health benefits, not only for respiratory conditions but also for cardiovascular outcomes. Other gaseous pollutants, such as O_3_ and CO, showed varying degrees of association with cardiovascular outcomes. The weaker associations observed for O_3_ might reflect its different spatial distribution and atmospheric processes (including reactions that lead to an inverse relationship with NO_2_), in addition to the relatively limited monitoring of this pollutant. Nonetheless, O_3_ should not be overlooked, as associations have been observed between O_3_ and cardiovascular disease in other regions of the world (49). Marked seasonal variations in this pollutant are observed and levels of this pollutant are likely to rise as global temperatures increase. For CO, our results here are consistent with previous meta-analyses linking short-term CO exposure to risk of myocardial infarction (50).

The effect of air pollutant exposure on mortality was stronger than that on hospital admissions. This pattern remained consistent across pollutants; mortality outcomes generally exceeded admission outcomes, especially for PM_2.5_ (3.84% vs 0.46% increase in risk) and NO_2_ (1.08% vs 0.62% increase in risk). Our observations may reflect several LIC and LMIC-specific challenges, including delayed healthcare-seeking behaviour driven by financial constraints and limited healthcare access (51), and the limited availability of advanced cardiac care facilities (52,53). However, the larger mortality effects observed in our analysis are based on considerably fewer studies (typically 2–3 studies per pollutant) compared with hospital admission outcomes (10–12 studies per pollutant), which could influence the reliability and precision of the mortality estimates. For PM_2.5_ mortality in particular, high heterogeneity was largely driven by a single Iranian study that reported substantially larger effects than others, meaning the pooled estimate should be interpreted with caution until further studies are available. The higher mortality effects might also indicate that air pollution in LMICs has more severe cardiovascular consequences, possibly due to a more susceptible population or synergistic interactions with other risk factors such as household air pollution, occupational exposures, climate factors and co-morbidities (54).

An interesting finding from our analysis was the differences in risk of hospital admissions between the two-size metrics of particulate matter, with PM_10_ demonstrating stronger associations with cardiovascular admissions (RR: 1.0191; 95% CI: 1.0017–1.0368) compared to PM_2.5_ (RR: 1.0046; 95% CI: 1.0025–1.0067) per 10 μg/m^3^ increase. Typically, PM_2.5_ shows stronger associations with cardiovascular outcomes than PM_10_ (55), in principle due to the greater penetration of these particles into the lung, their larger particle surface area and, in general, the higher content of harmful constituents of the sources that PM_2.5_ arises from. A few factors might explain the pattern observed. First, when standardising to equivalent mass increments (10 μg/m^3^), the relative toxicity comparison is influenced by the typical concentration ranges of these pollutants. Given that PM_10_ concentrations are generally higher and more variable in LMIC urban settings, a 10 μg/m^3^ increment represents a smaller proportional change in exposure for PM_10_ than for PM_2.5_, potentially making direct comparison of effect sizes challenging. Also, in many LMIC settings, the proportion of PM_10_ derived from sources such as road dust, traffic and construction activities may be greater than in HICs, reflecting industrial emissions that are prevalent in rapidly urbanising environments, although it should be recognised that these sources will also contribute to particles in the PM_2.5_ size range. The relative toxicity of the sources of PM in LICs are less well determined than that of urban PM in HIC settings and laboratory sources of combustion-derived PM (55). Thus, there is a need for better attribution of different source contributions, and atmospheric reactions that affect the air pollution mixture people are exposed to, to link to health outcomes, and subsequently support targeted air quality interventions.

Studies performed in Iran constituted the majority of the dataset and were concentrated in urban areas. However, this dominance raises questions about generalisability. Non-Iranian studies, though fewer, showed similar directions of association but tended to report slightly weaker effects for PM_2.5_ and PM_10_. Iran is also frequently affected by transboundary desert dust storms, which contribute substantially to ambient particulate matter and differ in toxicity from combustion-derived particles more typical of other urban environments (56). WHO has highlighted desert dust as a pollutant warranting separate long-term assessment (57). While the data from Iran is informative, it highlights the paucity of data from other regions. In particular, South Asia and sub-Saharan African regions, home to some of the world’s most polluted cities, are underrepresented. This geographic imbalance parallels gaps identified in the GBD study, which notes that 90% of air pollution-related deaths occur in LMICs, yet there is often a dearth of air pollution monitoring stations in low-income regions (58), especially sub-Saharan Africa (22). The few studies from these potentially high-burden regions, where air pollution characteristics and healthcare infrastructure could differ markedly, represent a critical data gap. Our review focused specifically on LICs and lower-middle-income countries, excluding upper-middle-income countries such as China, South Africa, and Brazil, where much of the recent growth in air pollution–CVD research has occurred. This deliberate distinction means that the geographic distribution of studies observed here reflects evidence gaps specific to the lowest-income settings, which previous reviews combining all low and middle-income countries may not have revealed. Also, our review focused on incident (hospitalisation and mortality) rather than prevalence of cardiovascular risk factors such as hypertension and diabetes. Since these risk factors are themselves influenced by air pollution, through both shared and independent mechanisms, including them alongside incident CVD events would risk double-counting of effects in a pooled analysis. Our focus on incident events also reflects a stronger causal inference framework, with clear temporal associations between air pollutant exposure and cardiovascular outcomes, and reduced potential for confounding.

The observed associations between air pollution and cardiovascular disease are supported by mechanistic studies (15,59). All the criteria air pollutants investigated can induce oxidative stress and inflammation in the lung, both of which can be propagated to the systemic circulation (15)_._ Several pollutants, but most notably PM, induce a range of cardiovascular impairments including endothelial dysfunction, increased blood pressure, changes to heart rhythm, increased cardiac susceptibility to ischaemia, enhanced blood clotting, impaired fibrinolysis, to name but a few (47). Exposure to air pollution also promotes lipid peroxidation, vascular inflammation and accelerates atherosclerosis, and ultrafine particles may promote atherothrombotic vascular disease by directly accessing the blood and accumulating at sites of vascular inflammation (60). All these mechanisms will increase the risk of developing CVD but also increase the risk of cardiovascular events that lead to the outcomes studied in the current review. CO, a product of incomplete combustion, binds to haemoglobin, reducing oxygen delivery and exacerbating cardiac ischemia (50,61), a mechanism that may be particularly detrimental in populations with pre-existing anaemia, prevalent in LICs (62). Future studies should ascertain the extent to which these mechanisms contribute to differences in the profile of cardiovascular diseases between LICs, LMICs and HICs, and if this may engender differences in susceptibility to specific air pollutants.

### 4.1 Limitations

#### 4.1.1 Limitations of meta-analysis

While our study provides much needed insights from LICs and LMICs, several limitations warrant consideration. First, significant heterogeneity among the included studies owing to varying methodologies contributes substantially to the variability of pooled estimates and suggests the need for caution in generalising findings. Second, we assumed many of the included studies used generalised linear models, which assume a linear relationship between exposure and outcome. This assumption may not always hold true, as the exposure-response relationship is often reported to be supralinear (less steep at very high pollution levels). Third, our standardisation to a 10 μg/m^3^ increment for all pollutants, while facilitating comparisons, may not optimally represent the typical exposure contrasts for each pollutant in LMIC settings. Finally, the predominance of short-term exposure studies in our analysis prevents an adequate assessment of the long-term cardiovascular consequences of chronic air pollution in these settings.

#### 4.1.2 Limitations of systematic review

Our systematic review also has limitations. Although we used comprehensive search strategies, we may have missed relevant studies published in non-English languages or in journals not indexed in the databases searched. The substantial geographic concentration of studies in Iran limits generalising of our findings to other LICs and LMICs with different pollution profiles, population characteristics, and healthcare systems. The relatively small number of studies reporting certain outcomes (particularly mortality) resulted in less precise pooled estimates for these endpoints. This review is limited to incident events (hospitalisation and mortality) rather than prevalence and risk factors to ensure results reflect a causal inference framework (as from study protocol).

### 4.2 Future research directions

Advancing research on air pollution and cardiovascular disease in LICs and LMICs requires a paradigm shift toward longitudinal, context-specific, and interdisciplinary approaches. Prioritising longitudinal cohort studies in regions with a high pollution burden will help to clarify the cumulative risks of chronic exposure in settings with a different socioeconomic status and healthcare disparities. It is highly likely that air pollution sources and PM composition will differ from HICs, yet assessment of this in LICs and LMICs (and between locations with different degrees of urbanisation) has been minimal. Source apportionment methodologies would help disentangle region-specific contributors such as industrial emissions, traffic, or agricultural burning, although such studies would require sufficient support to perform adequately. Not only would this information provide insight into which air pollutants are most harmful, but it would help in the design of targeted mitigation strategies to maximise potential health benefits for given resources. Population characteristics will differ between nations, including demographics that may be more vulnerable to air pollution exposure and health statuses that incur biological susceptibility to air pollutants. Mapping population demographics, even as far as profiling of gene variants that confer greater susceptibility, would contextualise air pollutant-outcome risk scores by clarifying the extent to which certain populations or individuals are biologically more susceptible to air pollution, thus enabling targeted public health interventions and more precise risk assessments.

## 5. Conclusion

This systematic review and meta-analysis found significant associations between ambient air pollution and CVD in LICs and LMICs, complementing the findings of studies in higher income regions. High variability was observed for risk ratios due to the relatively limited number of studies identified and the different approaches used within them. However, given the overall positive associations between several air pollutants and cardiovascular mortality and hospital admission for CVD, the high levels of air pollution, as well as the structural and health inequities observed in many LICs and LMICs, suggest that the burden of air pollution on cardiovascular health is likely to be substantial. While a growing body of evidence on air pollution and cardiometabolic risk factors exists in broader LMIC settings, substantial gaps remain in studies reporting clinical CVD events in the LIC. Thus, there is a clear need for action on air pollution in LICs and LMICs, in terms of providing the evidence base to establish the risks of different sources of air pollution that exert the greatest effect on health. Doing so would greatly aid the justification of resources to improve air quality through policies that will provide a concerted approach of ground-level intervention, public awareness and appropriate health care provision.

## Data Accessibility Statement

Data extracted for this meta-analysis, including summary estimates and data dictionary, will be available upon request to the corresponding author following publication. Requests should be accompanied by a research proposal detailing the intended use and require approval from the study authors, along with a signed data-access agreement. The study protocol is publicly available via PROSPERO (registration number: CRD42023484929).

## Additional File

The additional file for this article can be found as follows:

10.5334/gh.1545.s1 Supplementary Materials. Full search strategy, supplementary figures (S1–S5), supplementary table (S1), and risk of bias assessment details.

## References

[1] Boogaard H, Walker K, Cohen AJ. Air pollution: the emergence of a major global health risk factor. Int Health. 2019 Nov 13;11(6):417–21. DOI: 10.1093/inthealth/ihz078 31613318

[2] State of Global Air Report 2024. [cited 2025 Feb 18]. https://www.stateofglobalair.org/resources/report/state-global-air-report-2024

[3] GBD 2021 Risk Factors Collaborators. Global burden and strength of evidence for 88 risk factors in 204 countries and 811 subnational locations, 1990–2021: a systematic analysis for the Global Burden of Disease Study 2021. Lancet. 2024 May 18;403(10440):2162–203. DOI: 10.1016/S0140-6736(24)00933-4 38762324 PMC11120204

[4] Ambient (outdoor) air pollution. World Health Organization; 2024 [cited 2025 Jan 2]. https://www.who.int/news-room/fact-sheets/detail/ambient-(outdoor)-air-quality-and-health

[5] Health Effects Institute. Traffic-Related Air Pollution: A Critical Review of the Literature on Emissions, Exposure, and Health Effects; 2010 [cited 2025 Feb 19]. https://www.healtheffects.org/publication/traffic-related-air-pollution-critical-review-literature-emissions-exposure-and-health

[6] He X, Zhai S, Liu X, Liang L, Song G, Song H, et al. Interactive short-term effects of meteorological factors and air pollution on hospital admissions for cardiovascular diseases. Environ Sci Pollut Res Int. 2022 Sept;29(45):68103–17. DOI: 10.1007/s11356-022-20592-5 35532824

[7] de Bont J, Jaganathan S, Dahlquist M, Persson Å, Stafoggia M, Ljungman P. Ambient air pollution and cardiovascular diseases: An umbrella review of systematic reviews and meta-analyses. J Intern Med. 2022 June;291(6):779–800. DOI: 10.1111/joim.13467 35138681 PMC9310863

[8] Vaičiulis V, Venclovienė J, Miškinytė A, Ustinavičienė R, Dėdelė A, Kalinienė G, et al. Association between Outdoor Air Pollution and Fatal Acute Myocardial Infarction in Lithuania between 2006 and 2015: A Time Series Design. Int J Environ Res Public Health. 2023 Mar 3;20(5). DOI: 10.3390/ijerph20054549 PMC10002310 36901560

[9] Mustafic H, Jabre P, Caussin C, Murad MH, Escolano S, Tafflet M, et al. Main air pollutants and myocardial infarction: a systematic review and meta-analysis. JAMA. 2012 Feb 15;307(7):713–21. DOI: 10.1001/jama.2012.126 22337682

[10] Cramer J, Jørgensen JT, Hoffmann B, Loft S, Bräuner EV, Prescott E, et al. Long-Term Exposure to Air Pollution and Incidence of Myocardial Infarction: A Danish Nurse Cohort Study. Environ Health Perspect. 2020 May;128(5):57003. https://pubmed.ncbi.nlm.nih.gov/32438827/32438827 10.1289/EHP5818 PMC7263451

[11] Toubasi A, Al-Sayegh TN. Short-term Exposure to Air Pollution and Ischemic Stroke: A Systematic Review and Meta-analysis. Neurology. 2023 Nov 7;101(19):e1922–32. DOI: 10.1212/WNL.0000000000207856 37758483 PMC10662999

[12] Shah ASV, Lee KK, McAllister DA, Hunter A, Nair H, Whiteley W, et al. Short term exposure to air pollution and stroke: systematic review and meta-analysis. BMJ. 2015 Mar 24;350:h1295. DOI: 10.1136/bmj.h1295 25810496 PMC4373601

[13] Jia Y, Lin Z, He Z, Li C, Zhang Y, Wang J, et al. Effect of Air Pollution on Heart Failure: Systematic Review and Meta-Analysis. Environ Health Perspect. 2023 July;131(7):76001. https://pubmed.ncbi.nlm.nih.gov/37399145/37399145 10.1289/EHP11506 PMC10317211

[14] Shah ASV, Langrish JP, Nair H, McAllister DA, Hunter AL, Donaldson K, et al. Global association of air pollution and heart failure: a systematic review and meta-analysis. Lancet. 2013 Sept 21;382(9897):1039–48. DOI: 10.1016/S0140-6736(13)60898-3 23849322 PMC3809511

[15] Miller MR, Shaw CA, Langrish JP. From particles to patients: oxidative stress and the cardiovascular effects of air pollution. Future Cardiol. 2012 July;8(4):577–602. DOI: 10.2217/fca.12.43 22871197

[16] Franchini M, Mannucci PM. Short-term effects of air pollution on cardiovascular diseases: outcomes and mechanisms. J Thromb Haemost. 2007 Nov;5(11):2169–74. DOI: 10.1111/j.1538-7836.2007.02750.x 17958737

[17] Jaganathan S, Jaacks LM, Magsumbol M, Walia GK, Sieber NL, Shivasankar R, et al. Association of long-term exposure to fine particulate matter and cardio-metabolic diseases in low- and middle-income countries: A systematic review. Int J Environ Res Public Health. 2019 July 16 [cited 2025 Mar 10];16(14):2541. DOI: 10.3390/ijerph16142541 31315297 PMC6679147

[18] Guo J, Chai G, Song X, Hui X, Li Z, Feng X, et al. Long-term exposure to particulate matter on cardiovascular and respiratory diseases in low- and middle-income countries: A systematic review and meta-analysis. Front Public Health. 2023 Mar 28 [cited 2025 Mar 10];11:1134341. DOI: 10.3389/fpubh.2023.1134341 37056647 PMC10089304

[19] Wilkinson MJ, Garshick MS, Taub PR. Prevention and Treatment of Cardiovascular Disease: Nutritional and Dietary Approaches. Springer Nature. 2021. https://play.google.com/store/books/details?id=_Xs9EAAAQBAJ.

[20] Rippe JM. Lifestyle strategies for risk factor reduction, prevention, and treatment of cardiovascular disease. Am J Lifestyle Med. 2019 Mar-Apr;13(2):204–12. DOI: 10.1177/1559827618812395 30800027 PMC6378495

[21] Minja NW, Nakagaayi D, Aliku T, Zhang W, Ssinabulya I, Nabaale J, et al. Cardiovascular diseases in Africa in the twenty-first century: Gaps and priorities going forward. Front Cardiovasc Med. 2022 Nov 10;9:1008335. DOI: 10.3389/fcvm.2022.1008335 36440012 PMC9686438

[22] Adeoye M, Rahimzadeh S, Taylor S, Shrikhande S, Perel P, Shah A, et al. The impact of air pollution on cardiovascular health outcomes in African populations: A scoping review. JACC Adv. 2024 Dec;3(12):101371. DOI: 10.1016/j.jacadv.2024.101371 39817083 PMC11733974

[23] Newell K, Kartsonaki C, Lam KBH, Kurmi OP. Cardiorespiratory health effects of particulate ambient air pollution exposure in low-income and middle-income countries: a systematic review and meta-analysis. Lancet Planet Health. 2017 Dec;1(9):e368–80. DOI: 10.1016/S2542-5196(17)30166-3 29851649

[24] Page MJ, McKenzie JE, Bossuyt PM, Boutron I, Hoffmann TC, Mulrow CD, et al. The PRISMA 2020 statement: an updated guideline for reporting systematic reviews. BMJ (Clinical research ed). 2021 Mar 29 [cited 2025 Feb 19];372. DOI: 10.1136/bmj.n71 PMC8005924 33782057

[25] Wu K, Ho HC, Su H, Huang C, Zheng H, Zhang W, et al. A systematic review and meta-analysis of intraday effects of ambient air pollution and temperature on cardiorespiratory morbidities: First few hours of exposure matters to life. EBioMedicine. 2022 Dec;86:104327. DOI: 10.1016/j.ebiom.2022.104327 36323182 PMC9626385

[26] Department for Environment, Food and Rural Affairs. UK AIR Air Information Resource [Internet]. [cited 2025 Feb 19]. https://uk-air.defra.gov.uk/reports/cat06/0502160851_Conversion_Factors_Between_ppb_and.pdf

[27] Duval S, Tweedie R. Trim and fill: A simple funnel-plot-based method of testing and adjusting for publication bias in meta-analysis. Biometrics. 2000 June;56(2):455–63. DOI: 10.1111/j.0006-341x.2000.00455.x 10877304

[28] Sicard P, Khaniabadi YO, Perez S, Gualtieri M, De Marco A. Effect of O, PM and PM on cardiovascular and respiratory diseases in cities of France, Iran and Italy. Environ Sci Pollut Res Int. 2019 Nov;26(31):32645–65. DOI: 10.1007/s11356-019-06445-8 31576506

[29] Leili M, Nadali A, Karami M, Bahrami A, Afkhami A. Short-term effect of multi-pollutant air quality indexes and PM2.5 on cardiovascular hospitalization in Hamadan, Iran: a time-series analysis. Environ Sci Pollut Res Int. 2021 Oct;28(38):53653–67. https://link.springer.com/article/10.1007/s11356-021-14386-4 34036506 10.1007/s11356-021-14386-4

[30] Moradi M, Mokhtari A, Mohammadi MJ, Hadei M, Vosoughi M. Estimation of long-term and short-term health effects attributed to PM standard pollutants in the air of Ardabil (using Air Q + model). Environ Sci Pollut Res Int. 2022 Mar;29(15):21508–16. DOI: 10.1007/s11356-021-17303-x 34761318

[31] Soleimani Z, Darvishi Boloorani A, Khalifeh R, Griffin DW, Mesdaghinia A. Short-term effects of ambient air pollution and cardiovascular events in Shiraz, Iran, 2009 to 2015. Environ Sci Pollut Res Int. 2019 Mar;26(7):6359–67. DOI: 10.1007/s11356-018-3952-4 30617889

[32] Taheri M, Nouri F, Ziaddini M, Rabiei K, Pourmoghaddas A, Shariful Islam SM, et al. Ambient carbon monoxide and cardiovascular-related hospital admissions: A time-series analysis. Front Physiol. 2023 Mar 8;14:1126977. DOI: 10.3389/fphys.2023.1126977 36969582 PMC10031048

[33] Dastoorpoor M, Sekhavatpour Z, Masoumi K, Mohammadi MJ, Aghababaeian H, Khanjani N, et al. Air pollution and hospital admissions for cardiovascular diseases in Ahvaz, Iran. Sci Total Environ. 2019 Feb 20;652:1318–30. DOI: 10.1016/j.scitotenv.2018.10.285 30586817

[34] Saifipour A, Azhari A, Pourmoghaddas A, Hosseini SM, Jafari-Koshki T, Rahimi M, et al. Association between ambient air pollution and hospitalization caused by atrial fibrillation. ARYA Atheroscler. 2019 May;15(3):106–12. https://pubmed.ncbi.nlm.nih.gov/31452658/31452658 10.22122/arya.v15i3.1843 PMC6698081

[35] Tabaghi S, Sheibani M, Khaheshi I, Miri R, Haji Aghajani M, Safi M, et al. Associations between short-term exposure to fine particulate matter and acute myocardial infarction: A case-crossover study. Clin Cardiol. 2023 Nov;46(11):1319–25. DOI: 10.1002/clc.24111 37501642 PMC10642339

[36] Davoodabadi Z, Soleimani A, Pourmoghaddas A, Hosseini SM, Jafari-Koshki T, Rahimi M, et al. Correlation between air pollution and hospitalization due to myocardial infarction. ARYA Atheroscler. 2019 July;15(4):161–7. https://pubmed.ncbi.nlm.nih.gov/31819749/31819749 10.22122/arya.v15i4.1834 PMC6884733

[37] Phung D, Hien TT, Linh HN, Luong LMT, Morawska L, Chu C, et al. Air pollution and risk of respiratory and cardiovascular hospitalizations in the most populous city in Vietnam. Sci Total Environ. 2016 July 1;557-558:322–30. DOI: 10.1016/j.scitotenv.2016.03.070 27016680

[38] Khan R, Konishi S, Ng CFS, Umezaki M, Kabir AF, Tasmin S, et al. Association between short-term exposure to fine particulate matter and daily emergency room visits at a cardiovascular hospital in Dhaka, Bangladesh. Sci Total Environ. 2019 Jan 1;646:1030–6. DOI: 10.1016/j.scitotenv.2018.07.288 30235588

[39] Rahman MM, Begum BA, Hopke PK, Nahar K, Newman J, Thurston GD. Cardiovascular morbidity and mortality associations with biomass- and fossil-fuel-combustion fine-particulate-matter exposures in Dhaka, Bangladesh. Int J Epidemiol. 2021 Aug 30;50(4):1172–83. DOI: 10.1093/ije/dyab037 33822936 PMC8633660

[40] Nakhlé MM, Farah W, Ziadé N, Abboud M, Salameh D, Annesi-Maesano I. Short-term relationships between emergency hospital admissions for respiratory and cardiovascular diseases and fine particulate air pollution in Beirut, Lebanon. Environ Monit Assess. 2015 Apr;187(4):196. DOI: 10.1007/s10661-015-4409-6 25792024

[41] Tian Y, Liu H, Liang T, Xiang X, Li M, Juan J, et al. Ambient air pollution and daily hospital admissions: A nationwide study in 218 Chinese cities. Environmental pollution (Barking, Essex: 1987). 2018 Nov [cited 2025 Mar 17];242(Pt B). DOI: 10.1016/j.envpol.2018.07.116 30096542

[42] Tian Y, Liu H, Wu Y, Si Y, Song J, Cao Y, et al. Association between ambient fine particulate pollution and hospital admissions for cause specific cardiovascular disease: time series study in 184 major Chinese cities. BMJ (Clinical research ed). 2019 Dec 30 [cited 2025 Mar 17];367. DOI: 10.1136/bmj.l6572 PMC7190041 31888884

[43] Powell H, Krall JR, Wang Y, Bell ML, Peng RD. Ambient coarse particulate matter and hospital admissions in the Medicare cohort air pollution study, 1999–2010. Environmental health perspectives. 2015 Nov [cited 2025 Mar 17];123(11). DOI: 10.1289/ehp.1408720 PMC4629736 25872223

[44] Atkinson RW, Kang S, Anderson HR, Mills IC, Walton HA. Epidemiological time series studies of PM_2.5_ and daily mortality and hospital admissions: a systematic review and meta-analysis. Thorax. 2014 July [cited 2025 Mar 17];69(7):660–665. DOI: 10.1136/thoraxjnl-2013-204492 24706041 PMC4078677

[45] Lu F, Xu D, Cheng Y, Dong S, Guo C, Jiang X, et al. Systematic review and meta-analysis of the adverse health effects of ambient PM_2.5_ and PM_10_ pollution in the Chinese population. Environ Res. 2015 Jan;136:196–204. DOI: 10.1016/j.envres.2014.06.029 25460637

[46] Hayes RB, Lim C, Zhang Y, Cromar K, Shao Y, Reynolds HR, et al. PM_2.5_ air pollution and cause-specific cardiovascular disease mortality. International journal of epidemiology. 2020 Feb 1 [cited 2025 Mar 17];49(1). DOI: 10.1093/ije/dyz114 PMC7124502 31289812

[47] Miller MR, Newby DE. Air pollution and cardiovascular disease: car sick. Cardiovasc Res. 2020 Feb 1;116(2):279–94. DOI: 10.1093/cvr/cvz228 31583404

[48] Sagheer U, Al-Kindi S, Abohashem S, Phillips CT, Rajagopalan S, Kalra DK. Environmental pollution and cardiovascular disease: Part 1 of 2: Air pollution. JACC: Advances. 2024 Feb 1 [cited 2025 Apr 14]; DOI: 10.1016/j.jacadv.2023.100805 PMC11198409 38939391

[49] Zhang Z, Wang C, Lin C, Wu Y, Wei J, Lu J, et al. Association of long-term exposure to ozone with cardiovascular mortality and its metabolic mediators: evidence from a nationwide, population-based, prospective cohort study. Lancet Reg Health West Pac. 2024 Nov;52:101222. DOI: 10.1016/j.lanwpc.2024.101222 39444716 PMC11497431

[50] Lee KK, Spath N, Miller MR, Mills NL, Shah ASV. Short-term exposure to carbon monoxide and myocardial infarction: A systematic review and meta-analysis. Environment international. 2020 Oct [cited 2025 Apr 11];143. DOI: 10.1016/j.envint.2020.105901 32634667

[51] Peters DH, Garg A, Bloom G, Walker DG, Brieger WR, Rahman MH. Poverty and access to health care in developing countries. Ann N Y Acad Sci. 2008;1136:161–71. DOI: 10.1196/annals.1425.011 17954679

[52] Mocumbi AO. Cardiovascular Health Care in Low- and Middle-Income Countries. Circulation. 2024 Feb 20 [cited 2025 Feb 19]. DOI: 10.1161/CIRCULATIONAHA.123.065717 38377254

[53] Ragupathi L, Stribling J, Yakunina Y, Fuster V, McLaughlin MA, Vedanthan R. Availability, use, and barriers to cardiac rehabilitation in LMIC. Global Heart. 2017 Dec 1 [cited 2025 Feb 19];12(4):323–334. DOI: 10.1016/j.gheart.2016.09.004 28302548

[54] Chowdhury S, Pillarisetti A, Oberholzer A, Jetter J, Mitchell J, Cappuccilli E, et al. A global review of the state of the evidence of household air pollution’s contribution to ambient fine particulate matter and their related health impacts. Environ Int. 2023 Mar;173:107835. DOI: 10.1016/j.envint.2023.107835 36857905 PMC10378453

[55] Du Y, Xu X, Chu M, Guo Y, Wang J. Air particulate matter and cardiovascular disease: the epidemiological, biomedical and clinical evidence. J Thorac Dis. 2016 Jan;8(1):E8–19. DOI: 10.3978/j.issn.2072-1439.2015.11.37 26904258 PMC4740122

[56] Shahsavani A, Tobías A, Querol X, Stafoggia M, Abdolshahnejad M, Mayvaneh F, et al. Short-term effects of particulate matter during desert and non-desert dust days on mortality in Iran. Environ Int. 2020 Jan;134:105299. DOI: 10.1016/j.envint.2019.105299 31751828

[57] Lwin KS, Tobias A, Chua PL, Yuan L, Thawonmas R, Ith S, et al. Effects of desert dust and sandstorms on human health: A scoping review. GeoHealth. 2023 Mar 1 [cited 2025 Sept 23];7(3):e2022GH000728. DOI: 10.1029/2022GH000728 PMC9976568 36874170

[58] Fuller R, Landrigan PJ, Balakrishnan K, Bathan G, Bose-O’Reilly S, Brauer M, et al. Pollution and health: a progress update. Lancet Planet Health. 2022 June;6(6):e535–47. DOI: 10.1016/S2542-5196(22)00090-0 35594895 PMC11995256

[59] Miller MR. Oxidative stress and the cardiovascular effects of air pollution. Free Radic Biol Med. 2020 May 1;151:69–87. DOI: 10.1016/j.freeradbiomed.2020.01.004 31923583 PMC7322534

[60] Miller MR, Raftis JB, Langrish JP, McLean SG, Samutrtai P, Connell SP, et al. Inhaled nanoparticles accumulate at sites of vascular disease. ACS nano. 2017 May 23 [cited 2025 Apr 11];11(5). DOI: 10.1021/acsnano.6b08551 PMC5444047 28443337

[61] You J, Liu Y, Dong J, Wang J, Bao H. Ambient carbon monoxide and the risk of cardiovascular disease emergency room visits: a time-series study in Lanzhou, China. Environ Geochem Health. 2023 Nov;45(11):7621–36. DOI: 10.1007/s10653-023-01653-1 37395909

[62] GBD 2021 Anaemia Collaborators. Prevalence, years lived with disability, and trends in anaemia burden by severity and cause, 1990–2021: findings from the Global Burden of Disease Study 2021. Lancet Haematol. 2023 Sept;10(9):e713–34. DOI: 10.1016/S2352-3026(23)00160-6 37536353 PMC10465717

[63] Khaniabadi YO, Daryanoosh SM, Hopke PK, Ferrante M, De Marco A, Sicard P, et al. Acute myocardial infarction and COPD attributed to ambient SO2 in Iran. Environ Res. 2017 July;156:683–7. DOI: 10.1016/j.envres.2017.04.028 28477578

[64] Ghaffari S, Hajizadeh R, Pourafkari L, Shokouhi B, Tajlil A, Mazani S, et al. Air pollution and admissions due to ST elevation myocardial infarction-a time-series study from northwest of Iran. Environ Sci Pollut Res Int. 2017 Dec;24(35):27469–75. DOI: 10.1016/j.envres.2017.04.028 28980195

[65] Hosseinpoor AR, Forouzanfar MH, Yunesian M, Asghari F, Naieni KH, Farhood D. Air pollution and hospitalization due to angina pectoris in Tehran, Iran: a time-series study. Environ Res. 2005 Sept;99(1):126–31. DOI: 10.1016/j.envres.2004.12.004 16053936

[66] Le DN, Nguyen HAP, Ngoc DT, Do THT, Ton NT, Van Le T, et al. Air pollution and risk of respiratory and cardiovascular hospitalizations in a large city of the Mekong Delta Region. Environ Sci Pollut Res Int. 2022 Dec;29(60):91165–75. DOI: 10.1007/s11356-022-22022-y 35881281

[67] Vahedian M, Khanjani N, Mirzaee M, Koolivand A. Ambient air pollution and daily hospital admissions for cardiovascular diseases in Arak, Iran. ARYA Atheroscler. 2017 May;13(3):117–34. https://www.ncbi.nlm.nih.gov/pubmed/29147121 29147121 PMC5677328

[68] Siregar S, Idiawati N, Pan WC, Yu KP. Association between satellite-based estimates of long-term PM exposure and cardiovascular disease: evidence from the Indonesian Family Life Survey. Environ Sci Pollut Res Int. 2022 Mar;29(14):21156–65. DOI: 10.1007/s11356-021-17318-4 34750763

[69] Khaniabadi YO, Hopke PK, Goudarzi G, Daryanoosh SM, Jourvand M, Basiri H. Cardiopulmonary mortality and COPD attributed to ambient ozone. Environ Res. 2017 Jan;152:336–41. DOI: 10.1016/j.envres.2016.10.008 27842286

[70] Goudarzi G, Geravandi S, Foruozandeh H, Babaei AA, Alavi N, Niri MV, et al. Cardiovascular and respiratory mortality attributed to ground-level ozone in Ahvaz, Iran. Environ Monit Assess. 2015 Aug;187(8):487. DOI: 10.1007/s10661-015-4674-4 26141926

[71] Abdolahnejad A, Jafari N, Mohammadi A, Miri M, Hajizadeh Y, Nikoonahad A. Cardiovascular, respiratory, and total mortality ascribed to PM and PM exposure in Isfahan, Iran. J Educ Health Promot. 2017 Dec 4;6:109. DOI: 10.4103/jehp.jehp_166_16 29296610 PMC5747222

[72] Bonyadi Z, Ehrampoush MH, Ghaneian MT, Mokhtari M, Sadeghi A. Cardiovascular, respiratory, and total mortality attributed to PM_2.5_ in Mashhad, Iran. Environ Monit Assess. 2016 Oct;188(10):570. DOI: 10.1007/s10661-016-5574-y 27640165

[73] Hajizadeh Y, Jafari N, Mohammadi A, Momtaz SM, Fanaei F, Abdolahnejad A. Concentrations and mortality due to short- and long-term exposure to PM in a megacity of Iran (2014-2019). Environ Sci Pollut Res Int. 2020 Oct;27(30):38004–14. DOI: 10.1007/s11356-020-09695-z 32617810

[74] Mohammadian-Khoshnoud M, Habibi H, Manafi B, Safarpour G, Soltanian AR. Effects of air pollutant exposure on acute myocardial infarction. Heart Lung Circ. 2023 Jan;32(1):79–89. DOI: 10.1016/j.hlc.2022.10.009 36428180

[75] Motesaddi Zarandi S, Hadei M, Hashemi SS, Shahhosseini E, Hopke PK, Namvar Z, et al. Effects of ambient air pollutants on hospital admissions and deaths for cardiovascular diseases: a time series analysis in Tehran. Environ Sci Pollut Res Int. 2022 Mar;29(12):17997–8009. DOI: 10.1007/s11356-021-17051-y 34677770

[76] Sepandi M, Akbari H, Naseri MH, Alimohamadi Y. Emergency hospital admissions for cardiovascular diseases attributed to air pollution in Tehran during 2016–2019. Environ Sci Pollut Res Int. 2021 July;28(28):38426–33. DOI: 10.1007/s11356-021-13377-9 33733401

[77] Nabavi SM, Jafari B, Jalali MS, Nedjat S, Ashrafi K, Salahesh A. Environmental air pollution and acute cerebrovascular complications: an ecologic study in tehran, iran. Int J Prev Med. 2012 Oct;3(10):723–9. https://www.ncbi.nlm.nih.gov/pubmed/23112900 23112900 PMC3483001

[78] Kermani M, Goudarzi G, Shahsavani A, Dowlati M, Asl FB, Karimzadeh S, et al. Estimation of short-term mortality and morbidity attributed to fine particulate matter in the ambient air of eight Iranian cities. Ann Glob Health. 2018 Aug 31;84(3):408–18. DOI: 10.29024/aogh.2308 30835377 PMC6748288

[79] Nhung NTT, Schindler C, Chau NQ, Hanh PT, Hoang LT, Dien TM, et al. Exposure to air pollution and risk of hospitalization for cardiovascular diseases amongst Vietnamese adults: Case-crossover study. Sci Total Environ. 2020 Feb 10;703:134637. DOI: 10.1016/j.scitotenv.2019.134637 31731158

[80] Khaniabadi YO, Goudarzi G, Daryanoosh SM, Borgini A, Tittarelli A, De Marco A. Exposure to PM_10_, NO_2_, and O_3_ and impacts on human health. Environ Sci Pollut Res Int. 2017 Jan;24(3):2781–9. DOI: 10.1007/s11356-016-8038-6 27837472

[81] Bayat R, Ashrafi K, Shafiepour Motlagh M, Hassanvand MS, Daroudi R, Fink G, et al. Health impact and related cost of ambient air pollution in Tehran. Environ Res. 2019 Sept;176:108547. DOI: 10.1016/j.envres.2019.108547 31247432

[82] Gharehchahi E, Mahvi AH, Amini H, Nabizadeh R, Akhlaghi AA, Shamsipour M, et al. Health impact assessment of air pollution in Shiraz, Iran: a two-part study. J Environ Health Sci Eng. 2013 June 28;11(1):11. DOI: 10.1186/2052-336X-11-11 24499576 PMC3776287

[83] Toolabi A, Bonyadi Z, Ramavandi B. Health impacts quantification attributed to ambient particulate matter in the nearest Iranian city to the main dust source. Environ Monit Assess. 2022 Aug 12;194(9):666. DOI: 10.1007/s10661-022-10329-9 35962291

[84] Miri M, Ebrahimi Aval H, Ehrampoush MH, Mohammadi A, Toolabi A, Nikonahad A, et al. Human health impact assessment of exposure to particulate matter: an AirQ software modeling. Environ Sci Pollut Res Int. 2017 July;24(19):16513–9. DOI: 10.1007/s11356-017-9189-9 28555396

[85] Dastoorpoor M, Goudarzi G, Khanjani N, Idani E, Aghababaeian H, Bahrampour A. Lag time structure of cardiovascular deaths attributed to ambient air pollutants in Ahvaz, Iran, 2008–2015. Int J Occup Med Environ Health. 2018 July 4;31(4):459–73. DOI: 10.13075/ijomeh.1896.01104 29546882

[86] Downward GS, Hystad P, Tasmin S, Abe SK, Saito E, Rahman MS, et al. Long-term exposure to particulate matter and all-cause and cause-specific mortality in an analysis of multiple Asian cohorts. Environ Int. 2024 July;189(108803):108803. DOI: 10.1016/j.envint.2024.108803 38870578

[87] Jalali S, Karbakhsh M, Momeni M, Taheri M, Amini S, Mansourian M, et al. Long-term exposure to PM_2.5_ and cardiovascular disease incidence and mortality in an Eastern Mediterranean country: findings based on a 15-year cohort study. Environ Health. 2021 Oct 28;20(1):112. DOI: 10.1186/s12940-021-00797-w 34711250 PMC8555193

[88] Sadeghi M, Ahmadi A, Baradaran A, Masoudipoor N, Frouzandeh S. Modeling of the relationship between the environmental air pollution, clinical risk factors, and hospital mortality due to myocardial infarction in Isfahan, Iran. J Res Med Sci. 2015 Aug;20(8):757–62. DOI: 10.4103/1735-1995.168382 26664423 PMC4652309

[89] Javanmardi P, Morovati P, Farhadi M, Geravandi S, Khaniabadi YO, Angali KA, et al. Monitoring the impact of ambient ozone on human health using time series analysis and air quality model approaches. Fresenius Environ Bull. 2018;27(1):533–44.

[90] Miri M, Derakhshan Z, Allahabadi A, Ahmadi E, Oliveri Conti G, Ferrante M, et al. Mortality and morbidity due to exposure to outdoor air pollution in Mashhad metropolis, Iran. The AirQ model approach. Environ Res. 2016 Nov;151:451–7. DOI: 10.1016/j.envres.2016.07.039 27565880

[91] Brown PE, Izawa Y, Balakrishnan K, Fu SH, Chakma J, Menon G, et al. Mortality associated with ambient PM_2.5_ exposure in India: Results from the Million Death Study. Environ Health Perspect. 2022 Sept;130(9):97004. https://pmc.ncbi.nlm.nih.gov/articles/PMC9472672/36102642 10.1289/EHP9538 PMC9472672

[92] Shamsipour M, Hassanvand MS, Gohari K, Yunesian M, Fotouhi A, Naddafi K, et al. National and sub-national exposure to ambient fine particulate matter (PM_2.5_) and its attributable burden of disease in Iran from 1990 to 2016. Environ Pollut. 2019 Dec;255(Pt 1):113173. DOI: 10.1016/j.envpol.2019.113173 31521993

[93] Gurung A, Son JY, Bell ML. Particulate matter and risk of hospital admission in the Kathmandu Valley, Nepal: A case-crossover study. Am J Epidemiol. 2017 Sept 1;186(5):573–80. DOI: 10.1093/aje/kwx135 28911012

[94] Sarizadeh G, Jaafarzadeh N, Roozbehani MM, Tahmasebi Y, Moattar F. Relationship between the number of hospitalized cardiovascular and respiratory disease and the average concentration of criteria air pollutants (CAP) in Ahvaz. Environ Geochem Health. 2020 Oct;42(10):3317–31. DOI: 10.1007/s10653-020-00577-4 32367271

[95] Azimi F, Hafezi F, Ghaderpoori M, Kamarehie B, Karami MA, Sorooshian A, et al. Temporal characteristics and health effects related to NO_2_, O_3_, and SO_2_ in an urban area of Iran. Environ Pollut. 2024 May 15;349(123975):123975. DOI: 10.1016/j.envpol.2024.123975 38615834

[96] Akbarzadeh MA, Khaheshi I, Sharifi A, Yousefi N, Naderian M, Namazi MH, et al. The association between exposure to air pollutants including PM_10_, PM_2.5_, ozone, carbon monoxide, sulfur dioxide, and nitrogen dioxide concentration and the relative risk of developing STEMI: A case-crossover design. Environ Res. 2018 Feb;161:299–303. DOI: 10.1016/j.envres.2017.11.020 29178978

[97] Chen D, Mayvaneh F, Baaghideh M, Entezari A, Ho HC, Xiang Q, et al. Utilizing daily excessive concentration hours to estimate cardiovascular mortality and years of life lost attributable to fine particulate matter in Tehran, Iran. Sci Total Environ. 2020 Feb 10;703(134909):134909. DOI: 10.1016/j.scitotenv.2019.134909 31757557

