# Supplementary material for: Impact of Ambient Air Pollution on Cardiovascular Diseases in Low- and Lower-Middle-Income Countries: A Systematic Review and Meta-Analysis
Source: Glob Heart. 2026 Mar 30;21(1):31. doi: 10.5334/gh.1545 (PMC13045793; doi:10.5334/gh.1545)

Supplementary Material

1. **PRISMA Checklist** 2
2. **Search Strategy** 5
3. **Risk of Bias Assessment Full Description** 7
4. **Supplementary Tables and Figures** 8

Table S1 - Assessment for publication bias stratified by pollutant 8

Figure S1: Map of LICs and LMICs Included in the search terms based on the World Bank definition (2023) 9

Figure S2: Map of locations of studies in the meta-analysis 10

Figure S3: Forest plots for combined overall CVD effects 11

Figure S4: Sensitivity analysis - Forest plot of pooled relative risks for cardiovascular outcomes (excluding studies reporting Odds Ratios) 12

Figure S5: Forest plot of pooled relative risks for cardiovascular outcomes in a) LIC and LMIC countries excluding Iran which dominated the number of research studies (5 studies) b) Data from studies conducted in Iran only, for comparison (17 studies) 13

1. PRISMA Checklist

Section and Topic	Item #	Checklist item	Location where item is reported(page no)
**TITLE**			
Title	1	Identify the report as a systematic review.	1
**ABSTRACT**			
Abstract	2	See the PRISMA 2020 for Abstracts checklist.	2
**INTRODUCTION**			
Rationale	3	Describe the rationale for the review in the context of existing knowledge.	3-4
Objectives	4	Provide an explicit statement of the objective(s) or question(s) the review addresses.	4
**METHODS**			
Eligibility criteria	5	Specify the inclusion and exclusion criteria for the review and how studies were grouped for the syntheses.	4-5
Information sources	6	Specify all databases, registers, websites, organisations, reference lists and other sources searched or consulted to identify studies. Specify the date when each source was last searched or consulted.	4
Search strategy	7	Present the full search strategies for all databases, registers and websites, including any filters and limits used.	4
Selection process	8	Specify the methods used to decide whether a study met the inclusion criteria of the review, including how many reviewers screened each record and each report retrieved, whether they worked independently, and if applicable, details of automation tools used in the process.	5
Data collection process	9	Specify the methods used to collect data from reports, including how many reviewers collected data from each report, whether they worked independently, any processes for obtaining or confirming data from study investigators, and if applicable, details of automation tools used in the process.	5
Data items	10a	List and define all outcomes for which data were sought. Specify whether all results that were compatible with each outcome domain in each study were sought (e.g. for all measures, time points, analyses), and if not, the methods used to decide which results to collect.	5
	10b	List and define all other variables for which data were sought (e.g. participant and intervention characteristics, funding sources). Describe any assumptions made about any missing or unclear information.	5-7
Study risk of bias assessment	11	Specify the methods used to assess risk of bias in the included studies, including details of the tool(s) used, how many reviewers assessed each study and whether	5

		they worked independently, and if applicable, details of automation tools used in the process.	
Effect measures	12	Specify for each outcome the effect measure(s) (e.g. risk ratio, mean difference) used in the synthesis or presentation of results.	6
Synthesis methods	13a	Describe the processes used to decide which studies were eligible for each synthesis (e.g. tabulating the study intervention characteristics and comparing against the planned groups for each synthesis (item #5)).	6
	13b	Describe any methods required to prepare the data for presentation or synthesis, such as handling of missing summary statistics, or data conversions.	6
	13c	Describe any methods used to tabulate or visually display results of individual studies and syntheses.	7
	13d	Describe any methods used to synthesize results and provide a rationale for the choice(s). If meta-analysis was performed, describe the model(s), method(s) to identify the presence and extent of statistical heterogeneity, and software package(s) used.	6-7
	13e	Describe any methods used to explore possible causes of heterogeneity among study results (e.g. subgroup analysis, meta-regression).	6-7
	13f	Describe any sensitivity analyses conducted to assess robustness of the synthesized results.	6-7
Reporting bias assessment	14	Describe any methods used to assess risk of bias due to missing results in a synthesis (arising from reporting biases).	6
Certainty assessment	15	Describe any methods used to assess certainty (or confidence) in the body of evidence for an outcome.	7
**RESULTS**			
Study selection	16a	Describe the results of the search and selection process, from the number of records identified in the search to the number of studies included in the review, ideally using a flow diagram.	7
	16b	Cite studies that might appear to meet the inclusion criteria, but which were excluded, and explain why they were excluded.	7
Study characteristics	17	Cite each included study and present its characteristics.	7
Risk of bias in studies	18	Present assessments of risk of bias for each included study.	5
Results of individual studies	19	For all outcomes, present, for each study: (a) summary statistics for each group (where appropriate) and (b) an effect estimate and its precision (e.g. confidence/credible interval), ideally using structured tables or plots.	Table 1
Results of syntheses	20a	For each synthesis, briefly summarise the characteristics and risk of bias among contributing studies.	7-8

	20b	Present results of all statistical syntheses conducted. If meta-analysis was done, present for each the summary estimate and its precision (e.g. confidence/credible interval) and measures of statistical heterogeneity. If comparing groups, describe the direction of the effect.	Table 2
	20c	Present results of all investigations of possible causes of heterogeneity among study results.	7-9
	20d	Present results of all sensitivity analyses conducted to assess the robustness of the synthesized results.	7-9
Reporting biases	21	Present assessments of risk of bias due to missing results (arising from reporting biases) for each synthesis assessed.	8
Certainty of evidence	22	Present assessments of certainty (or confidence) in the body of evidence for each outcome assessed.	7-9
**DISCUSSION**			
Discussion	23a	Provide a general interpretation of the results in the context of other evidence.	9-12
	23b	Discuss any limitations of the evidence included in the review.	13
	23c	Discuss any limitations of the review processes used.	13
	23d	Discuss implications of the results for practice, policy, and future research.	13-14
**OTHER INFORMATION**			
Registration and protocol	24a	Provide registration information for the review, including register name and registration number, or state that the review was not registered.	4 & 16
	24b	Indicate where the review protocol can be accessed, or state that a protocol was not prepared.	16
	24c	Describe and explain any amendments to information provided at registration or in the protocol.	N/A
Support	25	Describe sources of financial or non-financial support for the review, and the role of the funders or sponsors in the review.	16
Competing interests	26	Declare any competing interests of review authors.	16
Availability of data, code and other materials	27	Report which of the following are publicly available and where they can be found: template data collection forms; data extracted from included studies; data used for all analyses; analytic code; any other materials used in the review.	16

2. Search Strategy

Cardiovascular disease terms: "Cardiovascular Diseases"[Mesh] OR "Heart Failure"[Mesh] OR "Stroke"[Mesh] OR "Myocardial Infarction"[Mesh]) OR "Cardiovascular Diseas*"[Title/Abstract] OR "Stroke"[Title/Abstract] OR "Myocardial Infarction"[Title/Abstract] OR "Heart Failure"[Title/Abstract]

Air Pollution terms: "Air Pollution"[Mesh] OR "Air Pollution, Indoor" [Mesh] OR "Air Pollutants" [Mesh:noexp] OR "Particulate Matter"[Mesh] OR "Coal Ash" [Mesh] OR "Dust" [Mesh] OR "Cosmic Dust" [Mesh] OR "Smog" [Mesh] OR "Smoke" [Mesh] OR "Soot" [Mesh] OR "Nitrogen Dioxide"[Mesh] OR "Ozone"[Mesh] OR "Sulfur Dioxide"[Mesh] OR "Ammonia"[Mesh]) OR ("Air Pollution"[Title/Abstract] OR "Air Pollutant*"[Title/Abstract] OR "Particulate Matter"[Title/Abstract] OR "Nitrogen Dioxide"[Title/Abstract] OR "Ozone"[Title/Abstract] OR "Sulfur Dioxide"[Title/Abstract] OR "Sulphur Dioxide"[Title/Abstract] OR "Ammonia"[Title/Abstract] OR " PM"[Title/Abstract] OR "PM10"[Title/Abstract] OR "PM2.5"[Title/Abstract] OR "PM2.5"[Title/Abstract] OR " Ultrafine particle*"[Title/Abstract] OR " Nitrogen dioxide"[Title/Abstract] OR "NO2"[Title/Abstract] OR "nitrogen oxide*"[Title/Abstract] OR "Ozone"[Title/Abstract] OR "Carbon monoxide"[Title/Abstract] OR "sulfur dioxide"[Title/Abstract] OR "Black carbon"[Title/Abstract] OR "Organic carbon"[Title/Abstract] OR "Ammonia"[Title/Abstract] OR " Ammonium"[Title/Abstract] OR " Organic dust "[Title/Abstract] OR "aerosol"[Title/Abstract] OR "inorganic dust"[Title/Abstract] OR " Volatile organic carbon "[Title/Abstract] OR "Semi-volatile organic carbon"[Title/Abstract] OR "traffic"[Title/Abstract] -

LIC and LMIC terms: "Developing Countries"[Mesh] OR "Developing Countries" [Title/Abstract] OR "low-income countr*"[Title/Abstract] OR “low income countr*”[Title/Abstract] OR "LIC" [Title/Abstract] OR "lower-middle-income countr*" [Title/Abstract] OR “lower middle income countr*”[Title/Abstract] OR "LMIC" [Title/Abstract] OR “Afghanistan” [Title/Abstract] OR “Burkina Faso” [Title/Abstract] OR “Burundi” [Title/Abstract] OR “Central African Republic” [Title/Abstract] OR “Chad” [Title/Abstract] OR “DR Congo” [Title/Abstract] OR “Eritrea” [Title/Abstract] OR “Ethiopia” [Title/Abstract] OR “Gambia” [Title/Abstract] OR “Guinea” [Title/Abstract] OR “Guinea-Bisseau” [Title/Abstract] OR “Liberia” [Title/Abstract] OR “Madagascar” [Title/Abstract] OR “Malawi” [Title/Abstract] OR “Mali” [Title/Abstract] OR “Mozambique” [Title/Abstract] OR “Niger” [Title/Abstract] OR “North Korea” [Title/Abstract] OR “Rwanda” [Title/Abstract] OR “Sierra Leone” [Title/Abstract] OR “Somalia” [Title/Abstract] OR “South Sudan” [Title/Abstract] OR “Sudan” [Title/Abstract] OR “Syria” [Title/Abstract] OR “Togo” [Title/Abstract] OR “Uganda” [Title/Abstract] OR “Yemen” [Title/Abstract] OR “Zambia” [Title/Abstract] OR “Afghanistan” [Mesh] OR “Burkina Faso” [Mesh] OR “Burundi” [Mesh] OR “Central African Republic” [Mesh] OR “Chad” [Mesh] OR “DR Congo” [Mesh] OR “Eritrea” [Mesh] OR “Ethiopia” [Mesh] OR “Gambia” [Mesh] OR “Guinea” [Mesh] OR “Guinea-Bisseau” [Mesh] OR “Liberia” [Mesh] OR “Madagascar” [Mesh] OR “Malawi” [Mesh] OR “Mali” [Mesh] OR “Mozambique” [Mesh] OR “Niger” [Mesh] OR “North Korea” [Mesh] OR “Rwanda” [Mesh] OR “Sierra Leone” [Mesh] OR “Somalia” [Mesh] OR “South Sudan” [Mesh] OR “Sudan” [Mesh] OR “Syria” [Mesh] OR “Togo” [Mesh] OR “Uganda” [Mesh] OR “Yemen” [Mesh] OR “Zambia” [Mesh] OR “Algeria” [Title/Abstract] OR “Angola” [Title/Abstract] OR “Bangladesh” [Title/Abstract] OR “Benin” [Title/Abstract] OR “Bhutan” [Title/Abstract] OR “Bolivia” [Title/Abstract] OR “Cambodia” [Title/Abstract] OR “Cameroon” [Title/Abstract] OR “Cape Verde” [Title/Abstract] OR “Comoros” [Title/Abstract] OR “Djibouti” [Title/Abstract] OR “Egypt” [Title/Abstract] OR “El Salvador” [Title/Abstract] OR “Eswatini” [Title/Abstract] OR “Ghana” [Title/Abstract] OR “Haiti” [Title/Abstract] OR “Honduras” [Title/Abstract] OR “India” [Title/Abstract] OR “Indonesia” [Title/Abstract] OR “Iran” [Title/Abstract] OR “Ivory Coast” [Title/Abstract] OR “Kenya” [Title/Abstract] OR “Kiribati” [Title/Abstract] OR “Kyrgyzstan” [Title/Abstract] OR “Laos” [Title/Abstract] OR “Lesotho” [Title/Abstract] OR “Mauritania” [Title/Abstract] OR “Micronesia” [Title/Abstract] OR “Mongolia” [Title/Abstract] OR “Morocco” [Title/Abstract] OR “Myanmar” [Title/Abstract] OR “Nepal” [Title/Abstract] OR “Nicaragua”

[Title/Abstract] OR “Nigeria” [Title/Abstract] OR “Pakistan” [Title/Abstract] OR “Palestine” [Title/Abstract] OR “Papua New Guinea” [Title/Abstract] OR “Philippines” [Title/Abstract] OR “Republic of the Congo” [Title/Abstract] OR “Samoa” [Title/Abstract] OR “Sao Tome and Principe” [Title/Abstract] OR “Senegal” [Title/Abstract] OR “Solomon Islands” [Title/Abstract] OR “Sri Lanka” [Title/Abstract] OR “Tajikistan” [Title/Abstract] OR “Tanzania” [Title/Abstract] OR “Timor-Leste” [Title/Abstract] OR “Tunisia” [Title/Abstract] OR “Ukraine” [Title/Abstract] OR “Uzbekistan” [Title/Abstract] OR “Vanuatu” [Title/Abstract] OR “Vietnam” [Title/Abstract] OR “Zimbabwe” [Title/Abstract] OR “Lebanon” [Title/Abstract] OR “Algeria” [Mesh] OR “Angola” [Mesh] OR “Bangladesh” [Mesh] OR “Benin” [Mesh] OR “Bhutan” [Mesh] OR “Bolivia” [Mesh] OR “Cambodia” [Mesh] OR “Cameroon” [Mesh] OR “Cape Verde” [Mesh] OR “Comoros” [Mesh] OR “Djibouti” [Mesh] OR “Egypt” [Mesh] OR “El Salvador” [Mesh] OR “Eswatini” [Mesh] OR “Ghana” [Mesh] OR “Haiti” [Mesh] OR “Honduras” [Mesh] OR “India” [Mesh] OR “Indonesia” [Mesh] OR “Iran” [Mesh] OR “Ivory Coast” [Mesh] OR “Kenya” [Mesh] OR “Kiribati” [Mesh] OR “Kyrgyzstan” [Mesh] OR “Laos” [Mesh] OR “Lesotho” [Mesh] OR “Mauritania” [Mesh] OR “Micronesia” [Mesh] OR “Mongolia” [Mesh] OR “Morocco” [Mesh] OR “Myanmar” [Mesh] OR “Nepal” [Mesh] OR “Nicaragua” [Mesh] OR “Nigeria” [Mesh] OR “Pakistan” [Mesh] OR “Palestine” [Mesh] OR “Papua New Guinea” [Mesh] OR “Philippines” [Mesh] OR “Republic of the Congo” [Mesh] OR “Samoa” [Mesh] OR “Sao Tome and Principe” [Mesh] OR “Senegal” [Mesh] OR “Solomon Islands” [Mesh] OR “Sri Lanka” [Mesh] OR “Tajikistan” [Mesh] OR “Tanzania” [Mesh] OR “Timor-Leste” [Mesh] OR “Tunisia” [Mesh] OR “Ukraine” [Mesh] OR “Uzbekistan” [Mesh] OR “Vanuatu” [Mesh] OR “Vietnam” [Mesh] OR “Zimbabwe” [Mesh] OR “Lebanon” [Mesh]

3. Risk of Bias Assessment Full Description

This evaluation covers three components: validity of the occurrence of CVD (rated 0 or 1), quality of air pollutant measurements (rated 0 or 1), and the degree of confounder adjustment (rated 0, 1, 2, or 3). In terms of CVD occurrence, we regarded a diagnosis to be valid if it was based on clinical, laboratory, and imaging data, or if it was classified by the International Classification of Diseases (a score of 0 was assigned in the absence of valid criteria). We evaluated the quality of air pollution measurements using parameters such as measurement frequency and the presence of missing data. If measurements were not performed daily or if there were more than 25% of missing data, a score of 0 was applied. In contrast, measurements taken at least daily with fewer than 25% of the data missing received a score of 1.

If no adjustments for seasonality, temperature, or long-term trends were carried, the confounder adjustment was assigned a score of 0. If only these three adjustments were performed, a score of 1 was assigned. A score of 2 was assigned for any additional adjustments for characteristics such as humidity or day of the week. Adjustments for influenza outbreaks and holidays, in addition to those equivalent to a score of 2, resulted in a score of 3. Furthermore, a study was classified good quality if it obtained the best possible score in each of the three components. However, if a single component earned a zero, the study was regarded as low quality. Studies that do not fit into either of the good or low quality were rated as average in quality.

Although not explicitly included in the scoring, we also took into account study design in the synthesis and interpretation.

4. Supplementary Tables and Figures

Table S1 - Assessment for publication bias stratified by pollutant

	SO_2	NO_2	NO	CO	O_3	PM_{10}	$PM_{2.5}$
Studies	16	15	3	14	15	19	18
Egger regression test P value	0.5664	0.0083	0.4905	< .0001	0.9773	0.5991	0.0861
RR (95% CI)	1.0015 (0.9986-1.0044)	1.0066 (1.0036-1.0097)	1.002 (0.9987-1.0053)	1.0001 (1.0001-1.0001)	0.9995 (0.9988-1.0002)	1.0168 (1.0017-1.0321)	1.0053 (1.0031-1.0075)
Adjusted RR (95% CI)	1.0013 (0.998-1.0047)	1.0064 (1.0039-1.0089)	1.001 (0.9925-1.0096)	1.0003 (1.0001-1.0006)	0.9971 (0.9887-1.0055)	1.0169 (1.0013-1.0328)	1.037 (1.0097-1.0651)
Number of studies adjusted	0	5	2	3	0	0	1

RR, relative risk; CI, confidence interval; $PM_{2.5}$, particulate matter ≤ 2.5 µm; PM_{10}, particulate matter ≤10 µm; NO_2, nitrogen dioxide; NO, nitric oxide; SO_2, sulphur dioxide; CO, carbon monoxide; O_3, ozone.

Figure S1: Map of LICs and LMICs Included in the search terms based on the World Bank definition (2023)

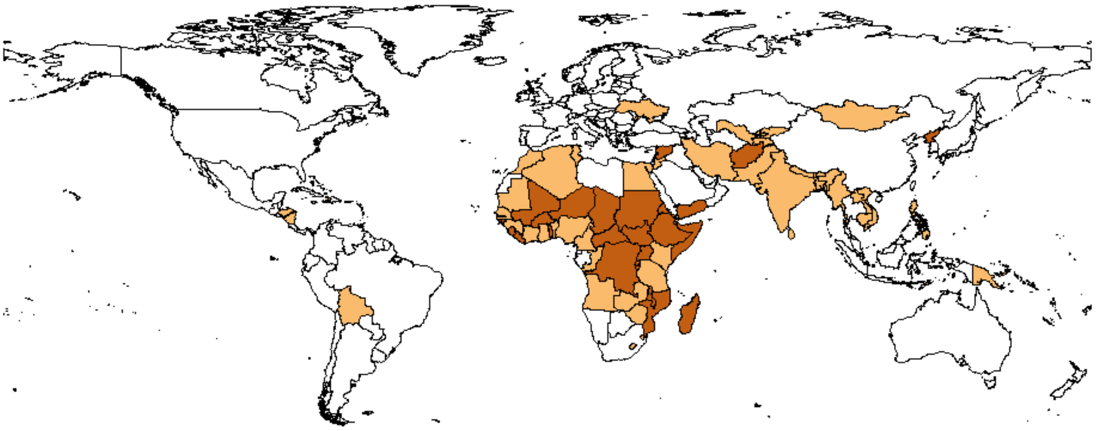

Dark Orange: Low-income countries

Light orange: Lower middle-income countries

Figure S2: Map of locations of studies in the meta-analysis

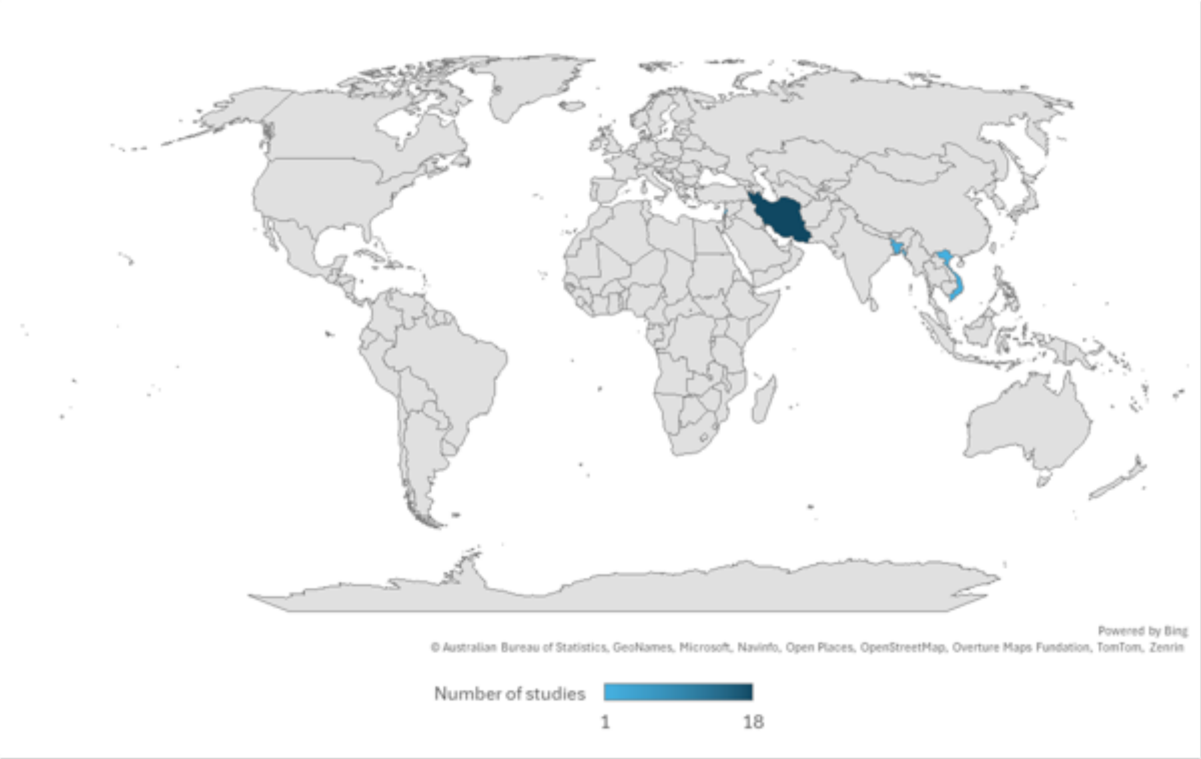

Figure S3: Forest plots for combined overall CVD effects

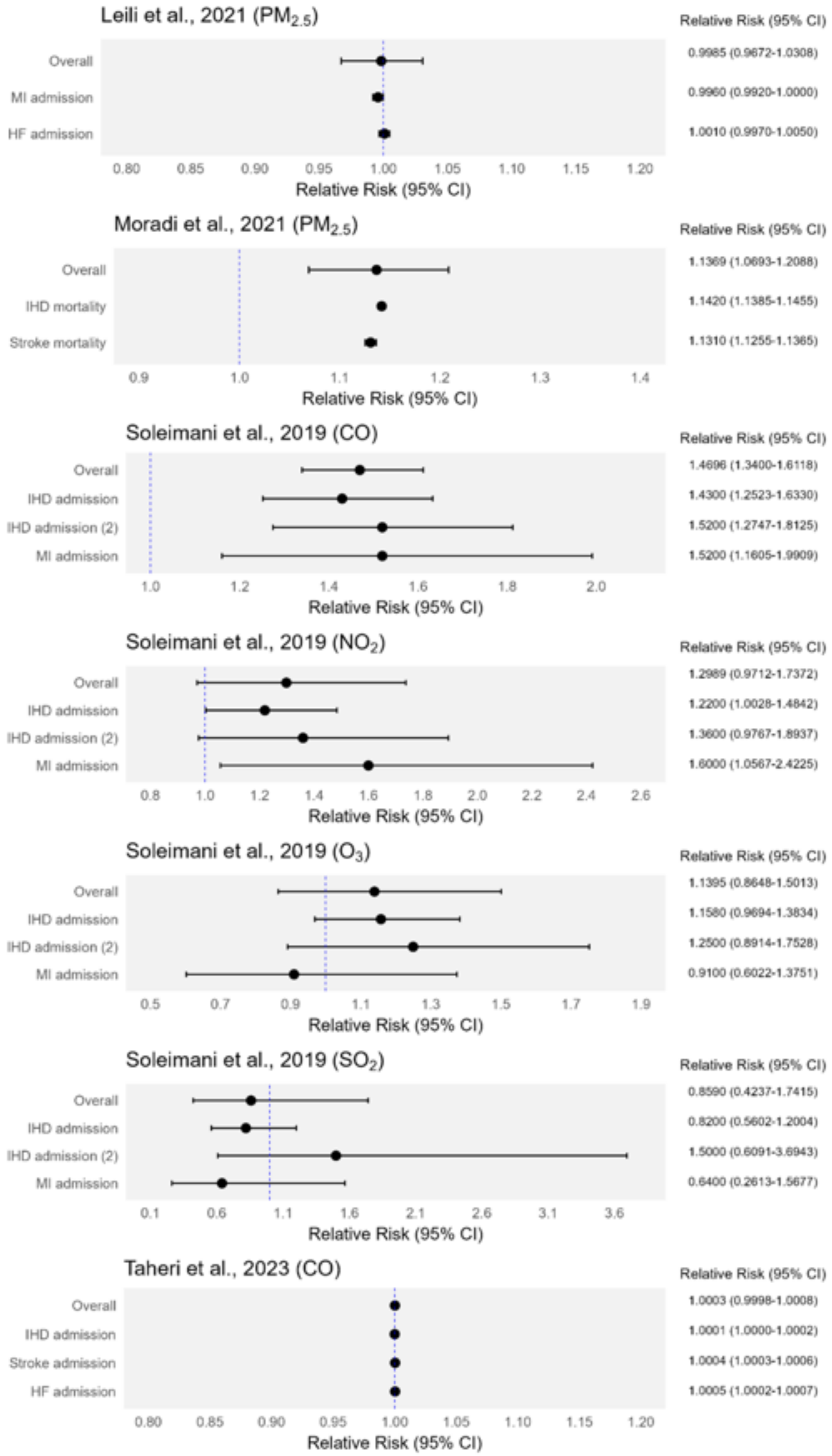

Figure S4: Sensitivity analysis - Forest plot of pooled relative risks for cardiovascular outcomes (excluding studies reporting Odds Ratios)

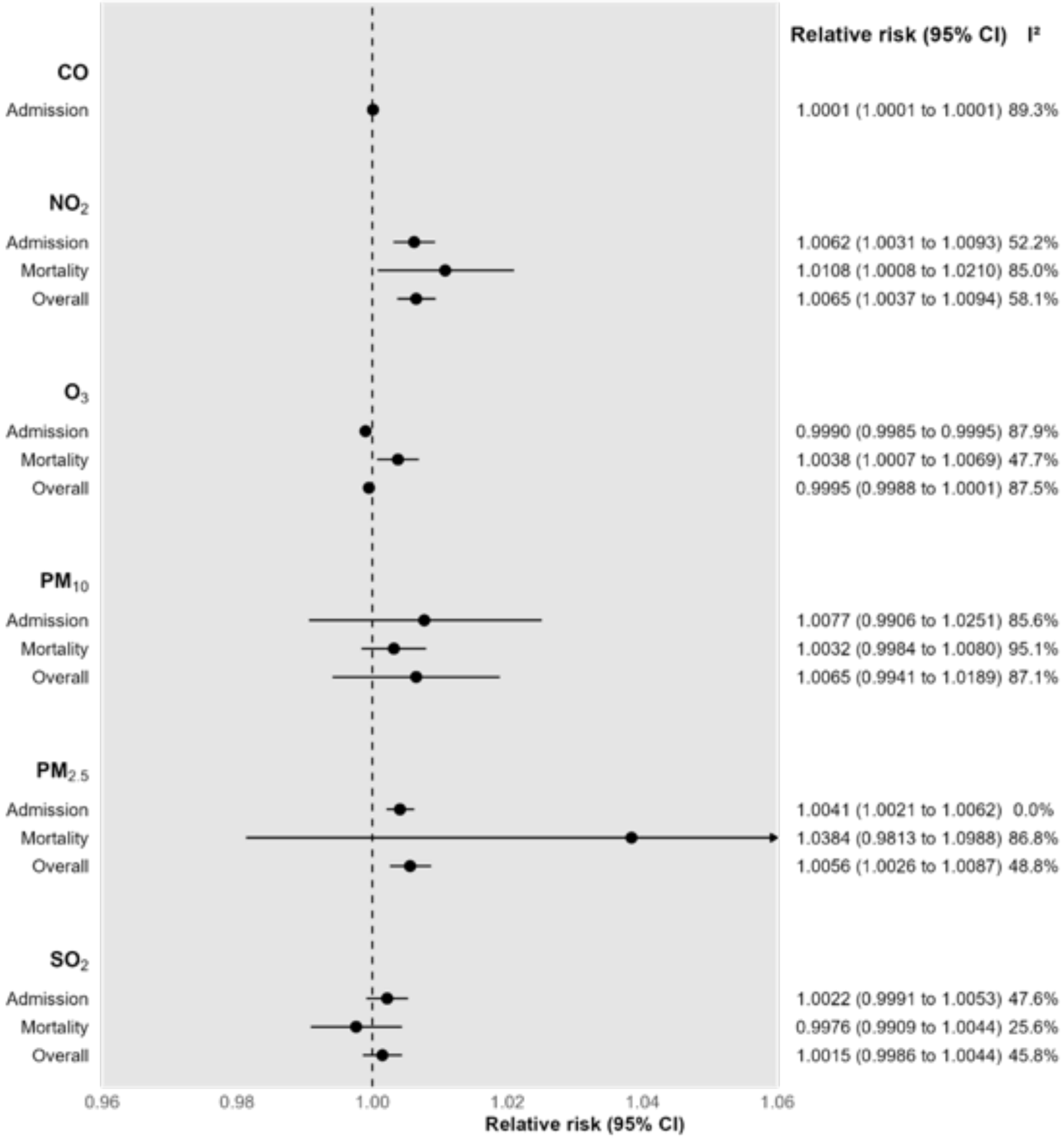

Figure S5: Forest plot of pooled relative risks for cardiovascular outcomes in a) LIC and LMIC countries excluding Iran which dominated the number of research studies (5 studies) b) Data from studies conducted in Iran only, for comparison (17 studies)

a)

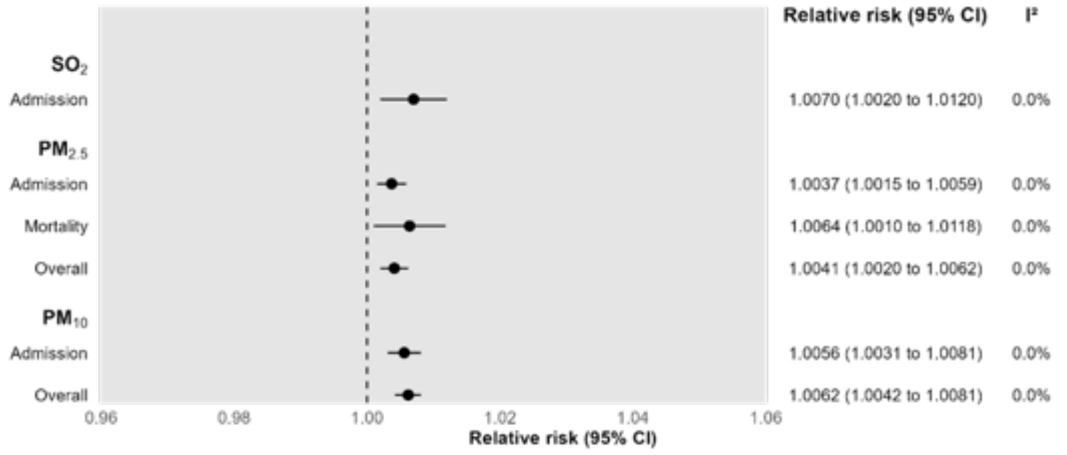

b)

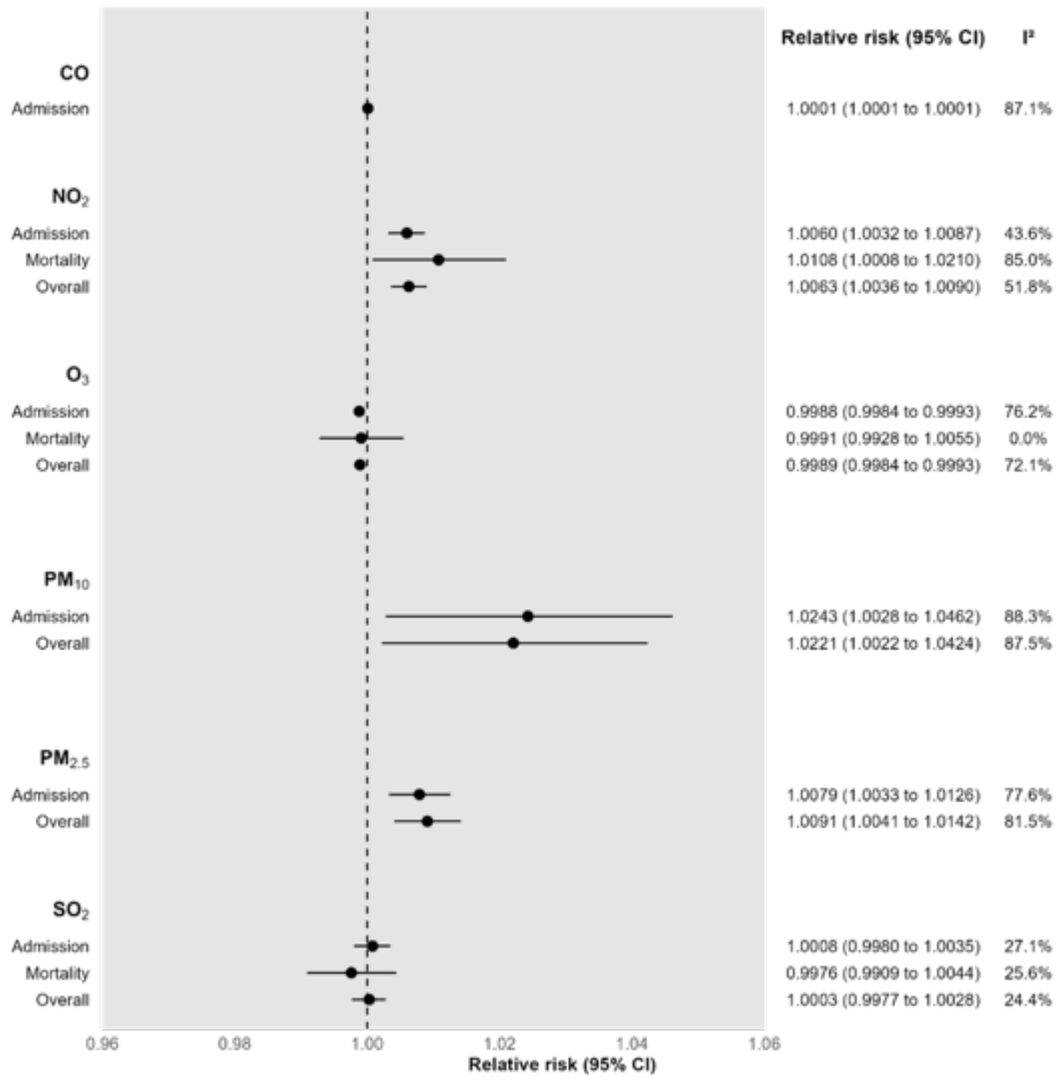

Supplement: Supplementary Materials. — Full search strategy, supplementary figures (S1–S5), supplementary table (S1), and risk of bias assessment details. [file gh-21-1-1545-s1.pdf]
